# Chip-Based Sensing of the Intercellular Transfer of Cell Surface Proteins: Regulation by the Metabolic State

**DOI:** 10.3390/biomedicines9101452

**Published:** 2021-10-13

**Authors:** Günter A. Müller, Matthias H. Tschöp, Timo D. Müller

**Affiliations:** 1 Institute for Diabetes and Obesity, Helmholtz Diabetes Center at Helmholtz Zentrum München, German Research Center for Environmental Health, Ingolstädter Landstraße 1, 85764 Oberschleissheim, Germany; timo.mueller@helmholtz-muenchen.de; 2 German Center for Diabetes Research, 85764 Oberschleissheim, Germany; matthias.tschoep@helmholtz-muenchen.de; 3 Department Biology I, Genetics, Ludwig-Maximilians-Universität München, 82152 Planegg-Martinsried, Germany; 4 Division of Metabolic Diseases, Department of Medicine, Technische Universität München, 81675 München, Germany; 5 Helmholtz Zentrum München, German Research Center for Environmental Health, 85764 Oberschleissheim, Germany

**Keywords:** cell-free chip-based assay, cell surface protein expression, glycosylphosphatidylinositol (GPI)-anchored proteins (GPI-APs), GPI-specific phospholipase D (GPLD1), insulin resistance, protein transfer

## Abstract

Glycosylphosphatidylinositol (GPI)-anchored proteins (GPI-APs) are anchored at the surface of mammalian blood and tissue cells through a carboxy-terminal GPI glycolipid. Eventually, they are released into incubation medium in vitro and blood in vivo and subsequently inserted into neighboring cells, potentially leading to inappropriate surface expression or lysis. To obtain first insight into the potential (patho)physiological relevance of intercellular GPI-AP transfer and its biochemical characterization, a cell-free chip- and microfluidic channel-based sensing system was introduced. For this, rat or human adipocyte or erythrocyte plasma membranes (PM) were covalently captured by the TiO_2_ chip surface operating as the acceptor PM. To measure transfer between PM, donor erythrocyte or adipocyte PM were injected into the channels of a flow chamber, incubated, and washed out, and the type and amount of proteins which had been transferred to acceptor PM evaluated with specific antibodies. Antibody binding was detected as phase shift of horizontal surface acoustic waves propagating over the chip surface. Time- and temperature-dependent transfer, which did not rely on fusion of donor and acceptor PM, was detected for GPI-APs, but not typical transmembrane proteins. Transfer of GPI-APs was found to be prevented by α-toxin, which binds to the glycan core of GPI anchors, and serum proteins in concentration-dependent fashion. Blockade of transfer, which was restored by synthetic phosphoinositolglycans mimicking the glycan core of GPI anchors, led to accumulation in the chip channels of full-length GPI-APs in association with phospholipids and cholesterol in non-membrane structures. Strikingly, efficacy of transfer between adipocytes and erythrocytes was determined by the metabolic state (genotype and feeding state) of the rats, which were used as source for the PM and sera, with upregulation in obese and diabetic rats and counterbalance by serum proteins. The novel chip-based sensing system for GPI-AP transfer may be useful for the prediction and stratification of metabolic diseases as well as elucidation of the putative role of intercellular transfer of cell surface proteins, such as GPI-APs, in (patho)physiological mechanisms.

## 1. Introduction

The expression of a specific set of cell surface proteins contributes to separation as well as exchange of substances and information between neighboring cells and between cells and surrounding milieu. Thereby, it plays tremendous roles in a multitude of cell biological processes, such as cell growth, differentiation and development, tissue and organ morphogenesis, as well as responsiveness of cells and tissues towards hormonal and environmental cues. In general, the tissue- and time-specific exposure of surface proteins is under cell-endogenous control and based on differential gene expression. The possibility of exogenous control, i.e., acquisition of cell surface proteins produced in contacting cells within the same tissue depot or in distinct cells of remote tissues or the blood compartment and transferred via the interstitial space or surrounding medium (e.g., body fluids, blood), respectively, has attracted less attention so far. The fusion of microvesicles budding from plasma membranes (PM) of donor cells [1,2,3] or of exosomes secreted from donor cells [4,5,6] and harboring a specific subset of membrane proteins with the PM of acceptor cells has been regarded as the typical molecular mechanism for the intercellular transfer of cell surface proteins.

Glycosylphosphatidylinositol-anchored proteins (GPI-APs) represent a specific class of cell surface proteins, which lack proteinaceous transmembrane domains and in humans encompass about 150 members ([7], Uniprot database). They are constituted by a hydrophilic protein moiety of variable size (1.5–200 kDa) and a glycosylphosphatidylinositol (GPI) moiety [8,9,10]. This amphiphilic GPI moiety consists of phosphatidylinositol and the core glycan, which is conserved from yeast to man and modified by additional glycan side chains [11]. It becomes post-translationally coupled via a phosphoethanolamine bridge and an amide bond to the carboxy-terminus of the protein moiety and mediates anchorage of GPI-APs at the PM by insertion of their fatty acyl chains into the outer leaflet of the lipid bilayer [12,13]. Cell surface anchorage by GPI confers some unique features to the protein moiety. Of particular relevance is the possibility of intercellular transfer (i.e., from the PM of donor cells to the PM of acceptor cells), which relies on the presence of the full-length GPI anchor (i.e., including its diacylglycerol/phosphatidate moiety) and the resulting biophysical consequences.

In fact, considerably less tight binding to and the more facile extraction from supported phospholipid/cholesterol mono- and bilayers of GPI-APs compared to transmembrane proteins has been demonstrated recently by a multitude of biophysical studies [14,15,16,17,18]. Moreover, two independent groups demonstrated less stable residence at PM of full-length GPI-APs compared to transmembrane proteins at a time point (more than 40 years ago) before the first identification of GPI anchors: Bouma and coworkers found that in course of incubation of cells and liposomes, certain membrane proteins, among them the GPI-AP acetylcholinesterase (AChE) are translocated from intact human erythrocytes to protein-free sealed liposomes in concert with the exchange of phospholipids, the original study object [19]. Medof and coworkers incubated purified human erythrocyte GPI-APs CD59 and CD55 or decay accelerating factor (DAF) in the detergent-solubilized state with sheep erythrocytes [20] and observed their tight association with erythrocyte membranes and in case of DAF maintenance of its biological activity [21]. These early findings have meanwhile been confirmed by other groups and extended to “empty” planar phospholipid bi- and monolayers and other cellular membranes [22,23,24,25,26,27,28,29]. In conclusion, full-length GPI-APs manage to translocate from detergent micelles into natural and artificial membranes and vice versa without loss of their biological function. In addition, more recent studies revealed (i) that a subset of full-length GPI-APs became released from the surface of rat adipocytes into incubation medium and into the blood of rats and humans in complex with (lyso)phosphatidylcholine and cholesterol in micelle-like structures [30,31] and (ii) that full-length GPI-APs become translocated from micelle-like complexes into rat adipocytes [32]. Remarkably, the efficacy of both release and translocation was strictly dependent on the metabolic state and age of the rats and humans [30,32,33]. This was reflected best in the correlation between both the serum level of full-length GPI-APs and the efficacy of their translocation into adipocytes and the blood glucose/plasma insulin levels in diabetic rats and human patients.

Importantly, step (i), the release of full-length GPI-APs with the complete GPI anchor retained from cellular donor membranes, has to be discriminated from the so-called shedding of GPI-APs which involves the proteolytic or lipolytic cleavage of their carboxy-terminus or GPI anchor, respectively. The resulting removal of the complete anchor moiety or diacylglycerol/phosphatidate portions causes liberation of a truncated soluble version, i.e., of the protein moiety only or the protein moiety with the glycan attached, of the GPI-APs from the PM [11,12,13]. Furthermore, step (ii), the translocation of full-length GPI-APs into cellular acceptor membranes, has to be discriminated from their intercellular transfer, as analyzed in the present study, which involves the simultaneous presence of donor and acceptor PM. Consequently, release of GPI-APs is prerequisite for their subsequent translocation which in combination exert the overall process, the intercellular transfer of full-length GPI-APs from mammalian “donor” cells to “acceptor” cells at the same or distant tissue depot(s) involving a paracrine and endocrine route, respectively. By nature, intercellular transfer of GPI-APs is prevented by shedding.

However, so far investigations have not addressed the potential (patho)physiological relevance of intercellular GPI-AP transfer and the following related issues: (i) transfer mechanism (spontaneous vs. regulated), (ii) components involved (GPI-APs, phospholipids, catalysts), (iii) factors determining transfer efficacy at the molecular (viscoelasticity and stiffness of PM) and physiological level (cell type, disease state). The limited interest in and knowledge about intercellular transfer of cell surface proteins, in general, and GPI-APs, in particular, as a putative mode for controlling their cell surface expression in multicellular organisms, in particular in disease states, presumably rely on the experimental expenditure for its analysis both in vitro and in vivo.

Here, a cell-free system for sensing of GPI-AP transfer under defined conditions of the donor and acceptor PM, including the surrounding incubation medium, is presented: PM derived from rat and human adipocytes or erythrocytes as donors of full-length GPI-APs were injected into the microfluidic channels of sensor chips, to which PM from rat and human adipocytes or erythrocytes had been immobilized as acceptors. After incubation with serum or factors putatively controlling the transfer and removal of the donor PM by washing, transfer of GPI-APs to acceptor PM was monitored by consecutive injection of antibodies against typical adipocyte and erythrocyte PM proteins. Incremental mass loading onto the chip caused by transferred proteins and bound antibodies triggered stepwise right-ward phase shifts of horizontal surface acoustic waves (SAW) propagating across the chip surface. Strikingly, mass loading was detected for GPI-APs but not for transmembrane proteins.

Taking cells and serum samples from genetically and diet-induced obese and diabetic rats, it was found that (i) both the donor and the acceptor PM are critical for the upregulation of GPI-AP transfer provoked by metabolic derangement in the obese and diabetic state, (ii) serum proteins interfere with GPI-AP transfer with those from metabolically deranged rats displaying higher potency compared to normal rats and (iii) the transfer involves non-membrane complexes of GPI-APs and lipids rather than membrane fusion. This sensing system may be useful for future delineation of the (patho)physiological roles of GPI-AP transfer as well as of the potential of GPI-AP transfer for the prediction and stratification of diseases. Together, the findings indicate that GPI-AP transfer may represent a mechanism for the extrinsic, i.e., not cell-autonomous, control of cell surface expression of proteins, and the coordination of the functional state of cells within the same or between different tissue depots. This (paracrine or endocrine) mechanism should be understood as transfer of biologically relevant matter, e.g., enzymes, binding proteins, rather than of information, e.g., signaling proteins, hormones, since the structure of the former but not of the latter is related to their function.

## 2. Materials and Methods

### 2.1. Materials

Human adipose derived stem cells (hADSC) were delivered by iXCells Biotechnologies (San Diego, CA, USA, Cat. Nr. 10HU-001). Partially purified phosphatidylinositol-specific phospholipase C (PI-PLC) from *Bacillus cereus* (1 unit/mg protein) was obtained from Sigma-Aldrich Chemie GmbH (Munich, Germany). Recombinant (*E. coli*) human glycosylphosphatidylinositol-specific phospholipase D1 (GPLD1) (0.5 units/mg protein) was purchased from Creative BioMart Inc. (New York, NY, USA). Anti-hCD73 antibody (mouse monoclonal, prepared against CD73 purified from human placenta with no cross-reactivity against mouse/rat CD73 [for sensing 1:1250; dot blotting 1:200]) was provided by Santa Cruz Biotechnology Inc., Heidelberg, Germany, (sc-32299). Anti-rCD73 antibody (rabbit polyclonal, affinity-purified, IgG isotype, prepared against recombinant full-size human CD73 with cross-reactivity against rat CD73 [for sensing 1:1500; for dot blotting 1:400]) was obtained from Genetex/Biozol (Eching, Germany). Antibodies against CD55 (ab231061, rabbit polyclonal, affinity-purified, IgG isotype, prepared against unconjugated His-tagged recombinant peptide corresponding to rat CD55 aa 254–372 and produced in *E. coli* [for sensing 1:2500] and ab253284, mouse monoclonal, protein G-purified from tissue culture supernatant, IgG2a, prepared against a recombinant fragment corresponding to the extracellular domain of human CD55 [for sensing 1:1000]), tissue-nonspecific alkaline phosphatase (TNAP) (ab95462, rabbit polyclonal, affinity-purified, IgG isotype, prepared against an unconjugated synthetic peptide corresponding to TNAP [for sensing 1:750; for dot blotting 1:250]), CD59 (ab248625, rabbit monoclonal, protein A-purified, IgG isotype, prepared against a synthetic peptide corresponding to human CD59 [for sensing 1:2500; for dot blotting 1:500], AChE (ab34533, goat polyclonal, multi-step purified, IgG isotype, biotinylated, prepared against purified AChE from bovine erythrocytes [for sensing 1:1500; for dot blotting 1:300]), annexin-V (ab54775, mouse monoclonal, tissue culture supernatant, IgG1, prepared against a recombinant full-length protein corresponding to human annexin-V aa 1–321 [for sensing 1:1800] and ab140068, rabbit polyclonal, immunogen affinity-purified, IgG, prepared against a recombinant protein corresponding to full-length annexin-V [for sensing 1:2500; for dot blotting 1:500]), anti-insulin receptor α-subunit (ab203037, rabbit polyclonal, protein A-purified, IgG, prepared against a synthetic peptide corresponding to human insulin receptor α aa 700–800 conjugated to keyhole limpet haemocyanin [for sensing 1:500] and ab283717, rabbit monoclonal, protein A-purified, IgG, prepared against a recombinant fragment from the rat insulin receptor α-subunit [for sensing 1:1500; for dot blotting 1:200]), Glycophorin-A (ab129024, rabbit monoclonal, protein A-purified, IgG, prepared against a synthetic peptide corresponding to a fragment of human glycophorin-A 100 aa distant from the carboxy-terminus [for sensing 1:750), Band-3 (ab108414, rabbit monoclonal, protein A-purified, IgG, prepared against a synthetic peptide corresponding to a fragment of rat Band-3 [for sensing 1:2000]), Glut4 (ab216661, rabbit polyclonal, protein A-purified, IgG, prepared against a synthetic peptide corresponding to aa 467–509 of mouse Glut4 [for sensing 1:2500; for dot blotting 1:500]), Glut1 (ab128033, rabbit polyclonal, immunogen affinity-purified, IgG, prepared against a synthetic peptide corresponding to aa 300–400 of mouse Glut1; ab14683, rabbit polyclonal, immunogen affinity-purified, IgG, prepared against a synthetic peptide corresponding to aa 481–492 of human Glut1 [for sensing 1:400; for dot blotting 1:150]), and Apo-AI (ab52945, rabbit monoclonal, protein A-purified, IgG, prepared against a synthetic peptide corresponding to aa 1–100 of human Apo-AI [for sensing 1:2000] and ab20453, rabbit polyclonal, immunogen affinity-purified, IgG, prepared against purified mouse Apo-AI from pooled mouse plasma high density lipoprotein [for sensing 1:2500]) were delivered by Abcam (Cambridge, UK). 1-ethyl-3-[3-dimethylaminopropyl]carbodiimide (EDC) and N-hydroxysulfosuccinimide (Sulfo-NHS, premium grade) were bought from Pierce/Thermo Scientific (Rockford, IL, USA). Protein A- and protein G-Sepharose (Cl-4B) were from Calbiochem/Merck (Darmstadt, Germany). Polystyrene Bio-Beads SM-2 (20–50 mesh) were bought from Bio-Rad Laboratories (Munich, Germany). NSB Reducer was obtained from GE Healthcare. *Ortho*-phenanthroline (Pha) was delivered by Sigma (Deisenhofen, Germany). Human blood and serum samples derived from the control probands of a previously approved, performed, and published study [32]. Other materials (highest purity available) were obtained as described previously [30,31,32,33].

### 2.2. Animal Handling

Male Wistar rats (Crl:WI(WU)) were obtained from Charles River (Sulzfeld, Germany). Rats were housed two per cage in an environmentally controlled room with a 12:12-h light–dark circle (light on at 06:00) and ad libitum access to water and standard rat chow (17.7 kJ/g, Ssniff diet R/M-H, V1535 with 18% (*w*/*v*) crude protein, 4.7% sugar, and 3.5% crude fat) (Ssniff, Soest, Germany). The rats, including their metabolic characterization, were made available by Sanofi Pharma Deutschland GmbH (Frankfurt am Main, Germany). Blood and serum samples were collected as reported previously [33].

### 2.3. Preparation of Rat Adipocytes from Epididymal Fat Pads

Primary rat adipocytes were prepared from epididymal fat pads of male Wistar rats (140–160 g, fed ad libitum) as described previously [30]. Finally, portions were suspended in 2.5 mL of adipocyte buffer (20 mM Hepes/KOH, pH 7.4, 140 mM NaCl, 4.7 mM KCl, 2.5 mM CaCl_2_, 1.2 mM MgSO_4_, 1.2 mM KH_2_PO_4_, 2% [*w*/*v*] BSA, 100 μg/mL gentamycin, 1 mM sodium pyruvate, 5.5 mM glucose) at 3.5 × 10^6^ cells/mL.

### 2.4. Differentiation and Culture of Human Adipocytes

Human adipose-derived stem cells (hADSCs) were isolated from lipoaspirate tissue from single normal donors collected during elective surgical liposuction procedures and cryopreserved at passage 1 (1.0 million cells/vial) by iXCells Inc., San Diego, CA, USA, Control hADSCs were demonstrated to be positive for CD29, CD44, CD73, CD90, and CD105 and to be negative for CD14, CD31, and CD45 and reported to differentiate into many different lineages including chondrogenic, osteogenic, neuronal, and adipogenic [34,35]. ADSCs were differentiated in vitro and further expanded for 3–4 passages as follows: The frozen cells were thawed by putting the vial in a 37 °C-water bath with gentle agitation for 1–2 min. The cells were transferred in a 15 mL conical tube with 5 mL of fresh ADSCs Growth Medium (iXCells Biotechnologies USA Inc., Cat. Nr. MD-0003) and then centrifuged (220× *g*, 5 min, 25 °C). After removal of the supernatant, the cells were re-suspended in fresh ADSCs Growth Medium and then cultured in one T75 flask with medium change every 2–3 days until the cells had reached 70–80% confluence. After removal of the medium, the cells were washed once with PBS (5 mL per flask). Following addition of 3–5 mL of 0.25% of trypsin-EDTA to the flask, the cells were incubated (5 min, 37 °C). Subsequent to neutralization of typsin-EDTA by adding 2–3 volumes of ADSCs Growth Medium, the cells were collected by centrifugation (220× *g*, 5 min, 25 °C) and then resuspended in the desired volume of medium. New culture flasks were seeded at 5 × 10^3^ cells/cm^2^ with medium change every 2–3 days until the cells had reached 70–80% confluence.

For adipocyte differentiation (12-well plate formate), hADSCs were grown in ADSCs Growth Medium to >95% confluence. After gentle aspiration of the medium using a pipet and replacement with 1.5 mL of fresh medium/well (at very slow rate to avoid cell detachment), the cells were grown for 2–3 days. The medium was aspirated and 1.5 mL of Adipocytes Differentiation Medium (iXCells Biotechnologies USA Inc., Cat. Nr. MD-0005) were added to the cells. The Adipocytes Differentiation Medium was changed every 3 days. The hADSCs were cultured in this medium for 10–14 days and then analyzed for the percentage of cells undergoing lipid droplet formation by Oil Red O-staining. Lipid droplets were observed in 7–10 days after adipogenic induction. hADSCs were regarded as differentiated human adipocytes when Oil Red-stained lipid droplets were detectable in more than 85% of the cells. After trypsinization, neutralization, and collection (see above), the human adipocytes were used for preparation of PM.

### 2.5. Preparation of Rat/Human Erythrocyte PM

Stripped erythrocyte membrane ghosts, which were mainly constituted of PM vesicles, were prepared from rat or human blood (acid-citrate-dextrose) as described previously [36]. Briefly, after centrifugation of the blood (outdated bank blood) and aspiration of the plasma and buffy layer, the packed cells were resuspended in PBS and then filtered through a column with a 5 cm bed prepared by mixing equal amounts of microcrystalline cellulose and α-cellulose for the removal of leukocytes and platelets. Thereafter, the erythrocytes were washed with PBS through the cellulose, then washed three times with ten volumes of PBS each, suspended in an equal volume of PBS and finally hemolysed, extracted, and washed by addition of ten volumes of 10 mM Tris/HCl (pH 7.6), 1 mM EGTA (TE). The suspension was centrifuged (12,000× *g*, 5 min, 22 °C). The pellet was resuspended in the same volume of TE. This washing cycle was repeated until complete elution of all hemoglobin (typically 4 times). The final pellet was suspended in 5 mM sodium phosphate buffer (pH 7.4) at 0.2 mg protein/mL and stored at −80 °C.

### 2.6. Preparation of Rat Adipocyte PM

PM were prepared from isolated rat adipocytes as described by Kiechle and coworkers [37], with minor modifications introduced previously [38]. Briefly, primary rat adipocytes (5 × 10^7^ cells) were washed and immediately homogenized in 2 mL of lysis buffer (25 mM Tris/HCl, pH 7.4, 0.5 mM EDTA, 0.25 mM EGTA, and 0.25 M sucrose, supplemented with 10 μg/mL leupeptin, 2 μM pepstatin, 10 μg/mL aprotinin, 5 μM antipain, and 200 μM PMSF) using a motor-driven Teflon-in-glass homogenizer (10 strokes with a loosely fitting pestle) at 22 °C. The defatted postnuclear infranatant obtained after centrifugation (1500× *g*, 5 min) was centrifuged (12,000× *g*, 15 min). The resulting pellet containing PM and mitochondria was resuspended in 10 mL of lysis buffer by hand homogenization and then fractionated on a discontinuous 25 mL sucrose gradient of 0.8 M, 1.06 M, and 2.02 M sucrose in 10 mM Mops/KOH (pH 7.4), 1 mM EDTA supplemented with protease inhibitor cocktail (see above). The gradient was centrifuged (24,000 rpm, 3 h, 4 °C, Beckman 25.1 rotor). The milky PM fraction at the interface between 0.8 M and 1.06 M sucrose was collected by suction and then diluted with 6 to 10 volumes of 10 mM Mops/KOH (pH 7.5), 1 mM EDTA, and then centrifuged (30,000× *g*, 20 min, 4 °C). The pellet was washed by suspending in 10 mL of lysis buffer by hand homogenization and again centrifuged. The pelleted PM were suspended in 10 mM Mops/KOH (pH 7.4), 0.25 M sucrose, 150 mM NaCl, 1 mM EDTA at 0.2 mg protein/mL, frozen in liquid N_2_, and stored until use at −80 °C.

### 2.7. Preparation of Human Adipocyte PM

Washed human adipocytes (5 × 10^7^ cells) differentiated from hADSCs were centrifuged (500× *g*, 5 min, 4 °C). The pelleted cells were suspended in cold PBS, again centrifuged, and after complete removal of the supernatant resuspended in 1 mL of buffer A (Invent Biotechnologies Inc., Plymouth, UK; Minute^TM^ Plasma Membrane Protein Isolation and Cell Fractionation Kit, Cat. Nr. SM-005). The adipocyte suspension was incubated (on ice, 10 min), vortexed vigorously (45 s), and then transferred to the filter cartridge. The cartridge was closed and centrifuged (16,000× *g*, 30 s, 4 °C, Eppendorf 5415C table top microcentrifuge). The pelleted adipocyte ghosts (in the course of having lost their lipid droplets during the centrifugation/filtration procedures) in the collection tube were resuspended with buffer A, then transferred to the same filter and centrifuged again (see above). The filter was discarded, and the adipocyte ghosts were resuspended in 0.5 mL of buffer A by vigorously vortexing (10 s). The adipocyte ghosts were centrifuged (700× *g*, 1 min, 4 °C). The supernatant was transferred to a fresh 1.5 mL microcentrifuge tube and then centrifuged (16,000× *g*, 30 min, 4 °C). After removal of the supernatant, the total PM fraction (typically 300–500 μg protein) was suspended in 250 μL of buffer B (Invent Biotechnologies Inc., Eden Prairie, MN, USA) by repeatedly pipetting up and down and vortexing and then centrifuged (7800× *g*, 10 min, 4 °C). The supernatant was transferred to a fresh 2-mL microfuge tube and supplemented with 1.6 mL of ice-cold PBS. After vigorous mixing, the suspension was centrifuged (16,000× *g*, 30 min, 4 °C). After removal of the supernatant, the pelleted PM (typically 150–200 μg protein) were suspended in 1 mL of 10 mM Mops/KOH (pH 7.5), 150 mM NaCl, 0.2 mM EGTA containing protease inhibitor cocktail (see above), and stored in liquid N_2_ until use.

### 2.8. Immobilization of PM at SAW Chip Surface by Ionic and Subsequent Covalent Capture

For ionic capture, uncoated negatively charged and highly hydrophilic TiO_2_ chips were used. Immobilization of erythrocyte/adipocyte PM containing positively charged, negatively charged, or zwitterionic phospholipids or combinations thereof with high efficacy was performed in the presence of 2 mM Ca^2+^ in 10 mM Hepes/NaOH (pH 7.5), 100 mM NaCl to enable salt bridges between the chip surface and the PM phospholipids [39]. PM (0.2 mg protein/mL) were injected at a flow rate of 25 μL/min for 4 min at 30 °C. After termination of the flow for 20 min at 30 °C, the chip was washed with 10 mM Hepes/NaOH (pH 7.5), 100 mM NaCl, and 2 mM EGTA at a flow rate of 150 μL/min for 20 min at 30 °C.

The measured phase shift elicited by binding of adipocyte PM was found to be considerably higher for TiO_2_-Ca^2+^ compared to Au and SiO_2_ chip surfaces (data not shown). Ca^2+^ easily covers the TiO_2_ surface forming a complete interactive layer. Thus, the PM phospholipids can bind to many sites on the surface at high density. In fact, high amounts of PM were found to be bound to the TiO_2_ surface indicating that close to complete coverage had been achieved. In contrast, Au and SiO_2_ surfaces were only partially covered, presumably due to repulsive forces between the bound PM, while other parts of the chip surface remained free of phospholipids (thereby forming a “mosaic”; data not shown). In addition, the presence of Ca^2+^ during the injection may prevent the repulsion between individual PM vesicles and trigger their fusion. Thus, capture of PM by the TiO_2_ chip surface possibly led to their transformation into flat supported membrane bilayers.

For subsequent covalent capture via the protein moieties of GPI-APs as well as the extracellular protein domains of adipocyte and erythrocyte membrane proteins, which resists Ca^2+^-removal during assaying GPI-AP transfer, the microfluidic channels of uncoated chips were primed by three injections of 250 μL, each, of immobilization buffer at a flow rate of 50 μL/min. Next, the chip surface was activated by a 250 μL injection of 0.2 M EDC and 0.05 M Sulfo-NHS (mixed from 2 × stock solutions right before injection) at a flow rate of 50 μL/min. After a waiting period of 3 min (flow rate 0) and subsequent washing of the channels with two 300 μL portions of PBS containing 0.5 mM EGTA (PBSE) at a flow rate of 180 μL/min, the residual activated groups on the chip surface were capped by injecting 200 μL of 1 M ethanolamine (pH 8.5) at a flow rate of 60 μL/min. Thereafter, the chips were washed two times with 125 μL of PBSE each at a flow rate of 150 μL/min and then two times with 160 μL of 10 mM Hepes/NaOH (pH 7.5) each at the same flow rate.

### 2.9. Determination of Transfer of GPI-APs from Donor to Acceptor PM by SAW Sensing

400 μL of rat or human adipocyte or erythrocyte donor PM (0.2 mg protein/mL) were injected (at 800–1200 s) at a flow rate of 60 μL/min into chips with rat or human erythrocyte or adipocyte acceptor PM consecutively immobilized by ionic and covalent capture. For initiation of transfer of GPI-APs from the donor PM presented in the chip microchannels as vesicles in solution to the acceptor PM immobilized at the chip TiO_2_ surface, the chips were incubated (1 h, from 1200 to 4800 s, 37 °C) at flow rate 0 (double hatched lines) in the absence or presence of certain agents for putative interference with transfer as indicated. For removal of the donor PM and any soluble or complex-bound GPI-APs from the microchannels, the chips were washed two times with 150 μL of PBSE each at a flow rate of 180 μL/min and then two times with 150 μL of 10 mM Hepes/NaOH, 150 mM NaCl (pH 7.5) (washing buffer) each at the same flow rate. Subsequently, for monitoring of the proteins transferred from the donor to the acceptor PM during the incubation, the protein composition of the captured acceptor PM was assayed by sequential injection of 75 μL of antibody against appropriate GPI-APs and transmembrane proteins (diluted as indicated in the Materials section) at a flow rate of 15 μL/min according to the order indicated in the figures (green and black arrows with hatched lines for initiation and termination of fluid flow, respectively). Finally, for demonstration of the nature of the transferred GPI-APs and their incorporation into the phospholipid bilayer of the acceptor PM, 75 μL of PI-PLC (*Bacillus cereus*, 5 ng) at a flow rate of 15 μL/min and then three portions of 220 μL of 0.1% (*w*/*v*) Triton X-100, 10 mM glycine (pH 12) at a flow rate of 200 μL/min, respectively, were injected.

For elucidation of the nature and amount of the GPI-APs and transmembrane proteins which became transferred to the acceptor PM in course of injection of the donor PM into the chip and incubation, the chip-integrated homogenous sensor system was used. It relies on the propagation of horizontal SAW along the chip surface which is affected by binding of any entities to the chip. This may happen either directly and unspecifically or via specific interaction partners immobilized onto the chip with the aid of ionic or covalent capturing procedures. The resulting right-ward phase shift of the SAW represents a measure for the loaded mass (i.e., presence and amount) brought about by entities of the sample analytes. Thus, both the initial capture of the acceptor PM by the TiO_2_ surface and the subsequent transfer of proteins from the injected donor PM to the captured acceptor PM was monitored in real-time as increases in phase shift. For this, the difference (∆) between the total phase shift provoked by all antibodies as a summation signal following injection of the last of the relevant antibodies and the phase shift left at the end of injection of PI-PLC (to correct for unspecific association of proteins, such as soluble GPI-APs or transmembrane proteins) was calculated as measure for the transfer of full-length GPI-APs for each donor–acceptor PM combination. The phase shift Δ is given upon correction for unspecific interaction (no acceptor PM) and normalization for varying capturing efficacy (of different chips for identical amounts of acceptor PM [32]).

### 2.10. Digestion with PI-PLC

For digestion of PM and proteins eluted from the chips according to ref. [40], 30 μL of PM (0.1 mg/mL), and 150 μL portions of eluate were supplemented with 50 μL and 10 μL, respectively, of 4-fold PIPLC buffer (80 mM Tris/HCl, pH 7.8, containing 0.4% (*w*/*v*) BSA, 600 mM NaCl, 2 mM EDTA, 4 mM DTT, and 0.4 mM PMSF) and then incubated (30 min, 30 °C) with partially purified PI-PLC from *Bacillus cereus* (0.25 mU and 0.1 mU, respectively).

### 2.11. Reconstitution of AChE and CD73 Detergent Micelles

bAChE and hCD73 prepared according to refs. [30,31] were lyophilized and then reconstituted into detergent micelles by suspending in 50 mM Tris/HCl (pH 7.4), 150 mM NaCl, 0.02% (*w*/*v*) NaN_3_ containing 15 mM octyl glucoside, and 0.1% (*w*/*v*) TX-100 at a final concentration of 10 µM, subsequent gentle vortexing, and final incubation (20 °C, 10 min). bAChE and hCD73 detergent micelles were immediately used.

### 2.12. Reconstitution of AChE/Band-3 and CD73/Glut4 Proteoliposomes

Proteoliposomes constituted of lysoPC, PC, cholesterol and bAChE, or hCD73 were prepared by mixing of these components in the detergent-solubilized state and subsequent removal of the detergent by adsorption onto polystyrene Bio-Beads according to previously published protocols [41,42,43] with modifications. This caused the spontaneous reconstitution of the components into unilamellar proteoliposomes. In detail, lysoPC (5 µmol), PC (0.25 µmol), PS (0.1 µmol), and cholesterol (0.5 µmol) in chloroform (10 mg/mL each) were dispersed together into a glass test tube in a total volume of 1 mL. After evaporation of the chloroform under a stream of N_2_ under atmospheric pressure, and then in a SpeedVac under high vacuum (60 min), the dried phospholipids (lipid films) were dispersed in 250 µL HSA (2 mg/mL) and subsequently completely dissolved by gentle vortexing and incubation (20 °C, 30 min). The hydrated lipid dispersion was subjected to six freezing–thawing cycles (−180 °C/+ 25 °C) and then passed 40 times through a polycarbonate membrane (0.2 µm) of a mini-extruder (Avanti Polar Lipids Inc., Alabaster, AL, USA). Reconstitution of bAChE was initiated by addition of 750 µL of 20 mM octyl glucoside and incubation (15 min, 25 °C; just for destabilization of the lipid bilayer). Subsequently, 100 µL of bAChE (0.3 nmol, freshly prepared from the lyophilized materials) or hCD73 (0.15 nmol) or 100 µL of rat adipocyte PM (solubilized by 0.1% (*w*/*v*) TX-100 as source for Glut4) or 100 µL of human erythrocyte PM (solubilized by 0.4% TX-100 as source for Band-3) were added to the mixture in a 1.5-mL microcentrifuge tube (Eppendorf Inc., Hamburg, Germany). Reconstitution was initiated by the addition of 50 mg damp Bio-Beads SM-2 to the tube and rotation on a tube rotator (20 rpm, 90 min, 20 °C). After addition of another 350 mg (damp weight) of Bio-Beads and rotation (180 min), the Bio-Beads were allowed to settle (5 min). The supernatant harboring 300 nM bAChE and 2.6 mM lipids in HSA (molar ratio = 8700:1) was carefully removed. For recovery, 200-µL portions of the supernatant were centrifuged (400,000× *g*, 1 h, 4 °C; Beckman TL-100 ultracentrifuge, TLA-100 rotor, 95,000 rpm). The pellets containing the proteoliposomes with reconstituted bAChE, hCD73, Glut4 or Band-3 were suspended in 100 µL of HSA (2 mg/mL). The proteoliposomes were sequentially sized through 0.4- and 0.2-µm polycarbonate membranes to select for large unilamellar ones (100–200 nm). Control liposomes were prepared by reconstitution of the lipids together with anchor-less bAChE or hCD73 (prepared by treatment of the purified GPI-APs with PI-PLC and subsequent recovery of the lipolytically cleaved versions from the detergent-depleted phase upon TX-114 partitioning) at the same ratios and using the same procedures as above.

### 2.13. TX-114 Partitioning

The sample (max. vol. 50 µL) was diluted to 150 µL with 10 mM Tris/HCl (pH 7.4), 150 mM NaCl, left on ice (5 min), then added to 600 μL of ice-cold 2.5% TX-114 (prepared by dissolving 37.5 g of TX-114 in 1 L of 10 mM Tris/HCl, pH 7.5, 150 mM NaCl on ice, precondensation at 37 °C, centrifugation, and use of the TX-114-enriched lower phase), mixed thoroughly and incubated (37 °C, 5 min) for induction of clouding according to ref. [44]. The detergent-enriched and depleted phases were separated by centrifugation (15,000× *g*, 2 min, 25 °C). The upper TX-114-depleted phase (100 µL) was removed without any disturbance of the interface, transferred to a new tube, and supplemented with TX-114 to a final concentration of 2.0% (*v*/*v*) for a second cycle of partitioning. After mixing and sequential incubation (0 °C, 5 min; 30 °C, 3 min), the solution was centrifuged (3000× *g*, 3 min). Thereafter, 100 µL of the supernatant were carefully transferred to a new tube avoiding any disturbance of the interface. This represented the final TX-114-depleted phase and was analyzed for the presence of the protein moieties of GPI-APs.

### 2.14. Adsorption of Eluate Materials to α-Toxin-Beads and Analysis by Dot Blotting

100 μL of chip eluate were added to 50 μL of PBS containing microspheres coupled to α-toxin in a 1.5-mL microcentrifuge tube. After vortexing, the mixtures were incubated (30 min, 22 °C) under head-over rotation. Subsequently, the tubes were placed into a magnetic separator and separation was allowed to occur for 30 to 60 s. The supernatants were removed and then the tube from the separator. The coupled microspheres were resuspended in 50 μL of PBS/TBN by vortexing and sonication for 20 s. The washing step with magnetic separation and resuspension was repeated three times with 100 μL of PBS each. Thereafter, the beads were suspended in 50 μL of PBS/TBN containing 2% (*w*/*v*) SDS, 20 mM DTT, and then incubated (95 °C, 5 min). The microspheres were again subjected to magnetic separation. The supernatant was removed and then immediately used for dot blotting.

For this, 10 μL portions (up to 8 replicates) of eluate, recombinant protein of interest (if available) and the corresponding primary antibodies were blotted onto PVDF membranes (Immuno-Blot PVDF Membrane, precut for minigels, Cat. Nr. 1620174; BIORAD, Munich, Germany). The membranes were incubated (25 °C, 2 h). Thereafter, the completely dry membranes were blocked with 5% (*w*/*v*) dry milk and 0.1% (*w*/*v*) BSA (fraction V, defatted) in 50 mM Tris/HCl (pH 7.4), 0.5 M NaCl, 0.05% (*w*/*v*) Tween-20 (TTBS) by incubation (25 °C, 2 h). The blocking buffer was poured off and the membranes were kept wet for the remainder of the procedure. The membranes were incubated (25 °C, 1 h) with appropriate antibodies in TTBS (diluted as indicated in the Materials section). Following washing of the membranes three times for 10 min each with sufficient volume of TTBS on a rocking water bath (25 °C), the membranes were incubated (25 °C, 2 h) with secondary antibodies coupled to horseradish peroxidase in TTBS. Following washing of the membranes three times for 10 min each with sufficient volume of TTBS on a rocking water bath (25 °C), the membranes were developed with ECL chemiluminescent detection kit (GE Healthcare, Braunschweig, Germany) according to the instructions of the manufacturer. Chemiluminescence of the dotted spots was quantitatively evaluated by phosphorimaging (Storm 840, Molecular Devices Inc., San Jose, CA, USA).

### 2.15. Statistical Analysis

All numerical data were presented as means ± standard deviations (SD). Statistical significance was calculated using GraphPad Prism6 software (version 6.0.2, GraphPad Software, San Diego, CA, USA) on the basis of either the two-tailed unpaired Student’s t-test between two experimental groups or the one-way ANOVA performed with Tukey’s post test for multiple comparisons. *p* ≤ 0.05 was considered to be significant.

### 2.16. Miscellaneous

Blood and serum samples were collected according to published procedures [30]. Preparation of Band-3 protein, bAChE, and hCD73, as well as recHDL and their reconstitution into liposomes, hCD73-recHDL, bAChE-recHDL, and micelle-like GPI-AP complexes, respectively, were described previously [32]. Pretreatment of serum (proteinase K digestion, PEG6000 precipitation, heat inactivation) was performed as described previously [32]. Chemical synthesis of PIG41, protein determination, preparation of α-toxin from the culture supernatant of *Clostridium septicum* and bAChE from bovine erythrocytes, coupling of α-toxin to Sepharose beads using conventional EDC/NHS-based protocol, SAW sensing with long-chain 3D CM-dextran sam^®^ 5 chips using a samX instrument (SAW/Nanotemper, Bonn/Munich, Germany) (Supplementary File S1) and evaluation were performed as has been described in detail previously [30,31,32,33,45]. Before injection of serum samples into CM-dextran chips, 0.1 vol. of 10 mg/mL carboxymethyl dextran (sodium salt, 0.15 M NaCl, 0.02% (*w*/*v*) NaN_3_ (NSB Reducer) was injected in order to reduce non-specific binding of sample components to the chip surface, and total cholesterol was determined with a colorimetric assay kit (Abcam, ab282928, Cambridge, UK).

## 3. Results

### 3.1. Chip-Based SAW Sensing Monitors the Transfer of Full-Length GPI-APs from Donor to Acceptor PM at Various Combinations, which Does Not Involve Membrane Fusion

**Figure 1:**
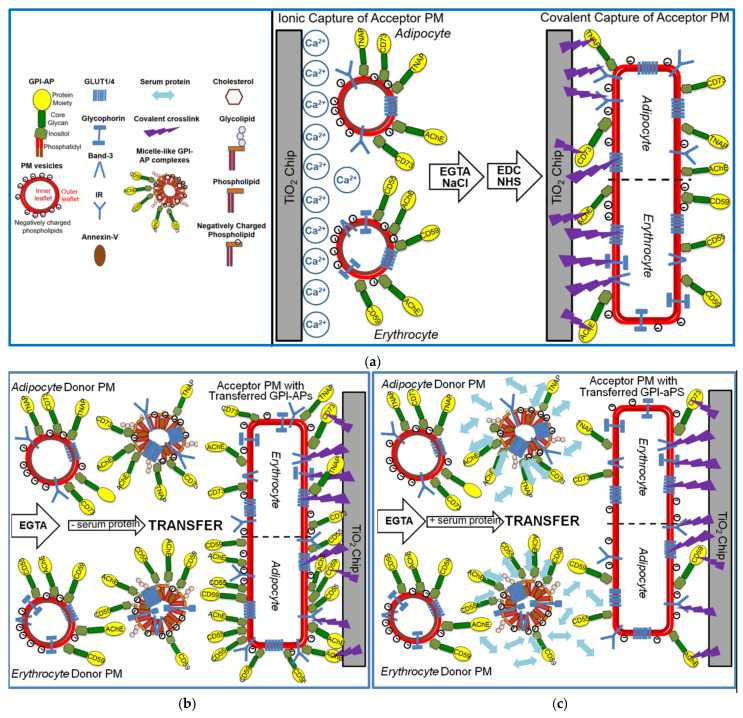

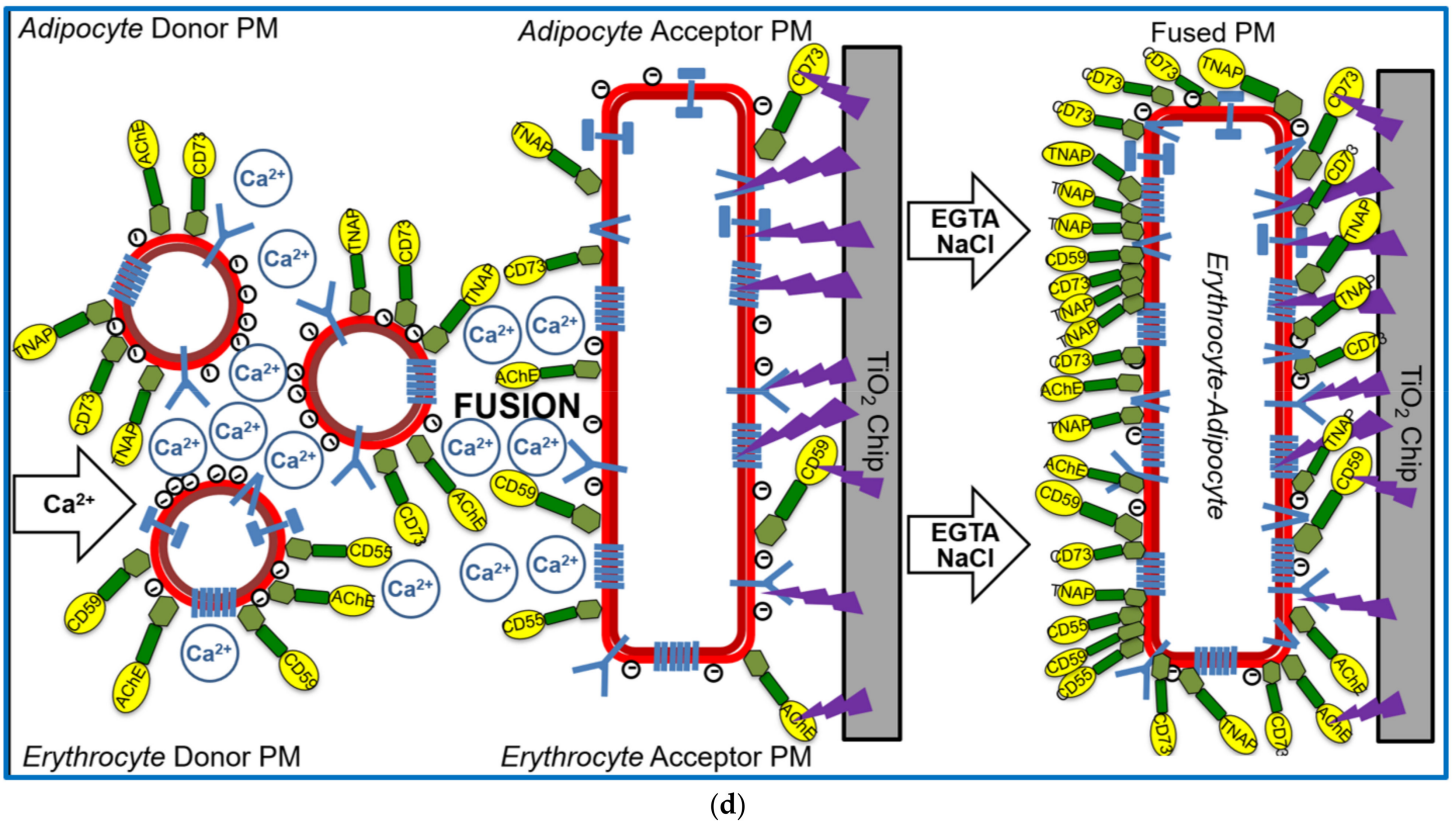
Model of the cell-free chip-based sensing system for analysis of transfer of GPI-APs between adipocyte and erythrocyte PM and the effect of serum proteins. (**a**) Ionic (middle panel) and covalent (right panel) capture of acceptor adipocyte and erythrocyte PM with legend for symbols (left panel). The possibility of formation of extended flat vesicular structures of PM at the chip surface in course of covalent capture is indicated. (**b**,**c**) Injection of adipocyte and erythrocyte donor PM together with EGTA in the absence (**b**) or presence (**c**) of serum proteins for analysis of transfer of GPI-APs to covalently captured adipocyte and erythrocyte acceptor PM via micelle-like GPI-AP complexes resulting in acceptor PM with transferred GPI-APs in the absence (**b**), but not in the presence (**c**), of serum proteins. The two combinations of donor and acceptor PM exhibiting the highest and lowest transfer efficacy for GPI-APs in the absence of serum proteins (**b**), hE → rE/hA (lower half) and h/rA → h/rE (upper half), respectively, are depicted (see also Table 1). (**d**) Injection of adipocyte and erythrocyte donor PM together with Ca^2+^ (left panel) for analysis of the fusion of adipocyte and erythrocyte donor and acceptor PM (middle panel). In all cases the TiO_2_ chips were sensed for GPI-APs and transmembrane proteins and the corresponding antibodies loaded onto the chip by measurement of SAW phase shift.

For set-up of an assay system reflecting the transfer of full-length GPI-APs between PM under defined conditions with regard to the type of the donor and acceptor cells, the incubation medium and any molecular entities affecting the transfer, a chip-based microfluidic sensor was established based on SAW. For this, the acceptor PM, derived either from primary rat adipocytes, human adipocytes differentiated from human adipose-derived stem cells (hADSC), or human erythrocytes, and harboring the GPI-APs acetylcholinesterase (AChE), tissue non-specific alkaline phosphatase (TNAP), 5’-nucleotidase (CD73), decay accelerating factor (CD55, DAF), and the complement membrane attack complex inhibitor (CD59), respectively, and in addition the transmembrane proteins, glucose transporter 4 and 1 (Glut4/1), insulin receptor (IR), Band-3, Glycophorin and Glut1, respectively in cell type-specific manner, were immobilized on the surface of TiO_2_ chips in course of a two-step capturing procedure (Figure 1a).

In the first step, acceptor PM (middle panel) were captured by negatively charged TiO_2_ chips in the presence of excess of Ca^2+^ through a combination of ionic (negatively-, and to a lower extent, positively charged phospholipids) and hydrophobic (zwitterionic phospholipids) interactions, yielding an almost complete coverage of the chip surface at high density and thereby increasing the efficacy of the subsequent covalent capture (right panel). In this second step, the acceptor PM were crosslinked to the activated TiO_2_ surface via the protein moieties of their constituent GPI-APs and transmembrane proteins using conventional EDC/NHS-based coupling chemistry with subsequent blocking of the reaction by ethanolamine. This resulted in chip channels with covalently captured and presumably enlarged and flattened PM vesicles (due to fusion in course of Ca^2+^-mediated absence of repulsive forces). Following removal of Ca^2+^ by EGTA and injection of NaCl to avoid fusion of the subsequently injected donor PM with the acceptor PM as well as their unspecific binding to the chip surface, respectively, the chips were ready for use as acceptor for GPI-APs in case of their putative transfer (right panel).

**Figure 2:**
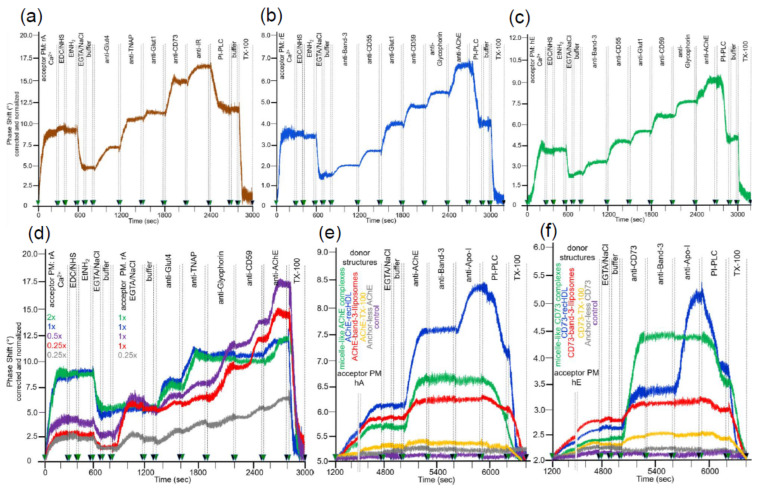
Capture of acceptor PM by SAW chip. PM from rat adipocytes (**a**,**d**), rat erythrocytes (**b**), human erythrocytes (**c**,**f**), and human adipocytes (**e**) were consecutively captured by chips via ionic (Ca^2+^) and covalent bonds (EDC/NHS, EtNH_2_; shown only for (**a**–**d**). (**a**–**c**) Characterization of the protein composition of the captured acceptor PM. Following consecutive injection of 150 µL of acceptor PM (0.15–0.20 mg protein/mL), 50 µL of EDC/NHS, 100 µL of 1 mM EGTA/2 M NaCl, and then 50 µL of buffer each at a flow rate of 30 µL/min, 75 µL of antibodies against GPI-APs and transmembrane proteins, then 75 µL of PI-PLC (*Bacillus cereus*, 7.5 mU/mL), subsequently 25 µL of washing buffer and finally 50 µL of TX-100 (0.1%) each at a flow rate of 15 µL/min were injected as indicated. (**d**) Demonstration of saturation of the chips with acceptor PM. After consecutive ionic and covalent capture of rat adipocyte PM (150 µL) at varying concentration (1× rel. concentration corresponded to 0.15–0.20 mg protein/mL) and then washing with EGTA/NaCl and subsequently washing buffer as performed for (**a**–**c**), 200 µL of fresh rat adipocyte acceptor PM were injected at the concentration (see above) indicated in the presence of 1 mM EGTA/2 M NaCl, followed by antibodies against GPI-APs and transmembrane proteins and finally TX-100 (0.1%) as performed for (**a**–**c**). (**e**,**f**) Translocation of GPI-APs from sources other than PM into acceptor PM. 100 µL of micelle-like bAChE complexes, AChE-recHDL, liposomes reconstituted with bAChE and Band-3, AChE reconstituted into TX-100 detergent micelles, anchorless AChE or buffer (control) were injected into chips with captured human adipocyte (**e**) or erythrocyte (**f**) PM (at 800–1200 s) at a flow rate of 15 µL/min. Thereafter at 1200 s, the flow rate was set at 0 for 1 h (hatched double line). Following injection of 200 µL of 1 mM EGTA/2 M NaCl and then 200 µL of washing buffer each at a flow rate of 120 µL/min, antibodies against GPI-APs and transmembrane proteins, subsequently PI-PLC and finally TX-100 (0.1%) were injected (see (**a**–**c**); but omission of final buffer injection) as indicated. (**a**–**f**) The measured phase shift is given upon correction for unspecific interaction (no acceptor PM) and normalization for variable capturing efficacy of different chips (i.e., for identical amounts of captured acceptor PM). The experiment was repeated once with similar results. Hatched lines with green and black arrows indicate start and termination, respectively, of cycles of fluid injection as indicated.

The efficacies of covalent capture of rat adipocyte and rat and human erythrocyte PM were monitored by an increase in right-ward phase shift (i.e., decrease in frequency) of the horizontal SAW propagating along the plane of the chip surface as measure for the mass of the loaded PM (Figure 2a–c). In each case, about 40 to 50% of the ionically captured PM (at 600 s) resisted injection of NaCl/EGTA and buffer, indicating covalent capture of a considerable portion of the PM (at 800 s). The nature of the captured PM was characterized by sequential injection (at 800 to 2700 s) of antibodies against typical GPI-APs and transmembrane proteins. The stepwise increases in phase shift reflecting antibody binding to the PM in sandwich demonstrated the differential expression of membrane proteins. Only a portion of the total phase shift increases (as summation signals) was due to GPI-APs. This was revealed by injection of bacterial PI-PLC (Figure 2a, 2400–2700 s; Figure 2b, 2700–2900 s), which specifically removed the diacylglycerol moiety from the GPI anchor. The resulting loss of the GPI-AP protein moieties, as well as of those PM vesicles captured through their GPI-APs from the chip, led to reduction in phase shift (Figure 2a–c). The remaining PI-PLC resistant component of the total phase shift increase (50 to 70%) was completely abrogated in course of injection of TX-100 (Figure 2a, at 2800–3000 s; Figure 2b, 3000–3200 s). This was compatible with disintegration of the structural integrity of the PM captured through their transmembrane proteins and/or those GPI-APs which escape cleavage by bacterial PI-PLC (possibly due to steric hindrance of PI-PLC access by the bound antibodies). For differentiation between the possibilities, the experiment with captured rat adipocyte and erythrocyte PM (Figure 2a,b) was modified with injection of PI-PLC prior to, rather than following, the injections of antibodies against GPI-APs and transmembrane proteins. Compatible with transmembrane anchorage, the phase shift increases in response to anti-Glut4, anti-Glut1, anti-IR, anti-Band-3, and anti-Glycophorin antibodies (data not shown) were very similar to those generated in the absence of PI-PLC injection (see Figure 2a,b). In contrast, the phase shift increases induced by anti-GPI-APs antibodies were considerably lower when injected following (data not shown) rather than prior to injection of PI-PLC (see Figure 2a,b). Remarkably, the GPI-APs analyzed differed in their susceptibility towards lipolytic cleavage, with the PI-PLC-induced phase shift decrease being most pronounced for TNAP and CD59, followed by CD73 and CD55, and lowest for AChE. Remarkably, PI-PLC exerted considerably less phase shift decrease with erythrocyte compared to adipocyte GPI-APs. Partial or complete resistance towards bacterial PI-PLC activity has been amply documented for a number of GPI anchors in the past and attributed to covalent modification of their core glycan (e.g., acylation of the 2-position of *myo*-inositol, which seems to be prevalent in erythrocyte GPI-APs [46,47,48]) or steric hindrance due to high packaging density of those GPI-APs (e.g., nanoclustering and oligomerization at PM microdomains or lipid rafts [49,50,51]) or tight interaction with other cell surface components [52]. Nevertheless, on basis of the moderate overall non-cleavability of the GPI-APs at the adipocyte and erythrocyte PM, which accounts for only 11 to 14% and 48 to 53%, respectively, based on the phase shift left upon correction for the transmembrane proteins, consecutive injections of PI-PLC and TX-100 were routinely performed to delineate the nature of the GPI-APs and transmembrane proteins, respectively, in the following transfer experiments.

The amount of PM amenable to capture by ionic and covalent bonds was saturatable, as revealed (Figure 2d) by the concentration-dependent increases in phase shift (at 0–300 s) and confirmed by subsequent anti-Glut4 and anti-TNAP injections (at 1300–1900 s) to up to a maximal value (shown only for rat adipocyte PM). Furthermore, only chips harboring submaximal amounts of covalently captured rat adipocyte PM (0.25×, 0.5× rel. concentration) displayed additional phase shift increases upon subsequent injection of rat adipocyte PM (at 800–1200 s) at submaximal (0.25× rel. concentration) to up to saturating (1× rel. concentration) amounts and subsequent anti-Glycophorin, anti-CD59, and anti-AChE injections (at 1900–2800 s).

Saturation was apparently due to complete coverage of the surface area with PM, i.e., exhaustion of the maximal capturing capacity, rather than to overriding of the measuring range of the sensing element of the chip. This has been revealed during previous titration experiments based on the capture of (increasing amounts of) purified GPI-APs by α-toxin-coated chips. The maximal phase shift increases obtained thereby were considerably higher than those elicited upon injection of (increasing amounts of) PM for ionic/covalent capture (Müller, G.A.; Ussar, S.; Müller, T.D.; unpublished data). This can be explained best by mutual steric hindrance of the PM vesicles during capturing, in combination with lower mass loading provoked by the PM (as assembly of lipids and proteins) compared to proteins exclusively. Under conditions of subsaturating capture of acceptor PM, the unspecific binding of full-length GPI-APs embedded in micelle-like complexes as prevalent in rat and human serum samples [32] was reduced to less than 8 and 5%, respectively, of the acceptor PM-induced phase shift by injection of NSB reducer plus 2 M NaCl prior to sample injection (according to manufacturers’ instructions) as outlined in the Materials and Methods section. Apparently, the conditions for the initial (prior to covalent) capture of acceptor PM did not support unspecific binding of full-length GPI-APs to chip. This presumably relied on ionic rather than hydrophobic interaction of the vesicular phospholipids with the TiO_2_ surface in combination with prevention of both ionic and hydrophobic interactions of the fatty acids of the GPI anchor and GPI-AP protein moiety by NaCl plus NSB, respectively.

Next, the suitability of the covalently captured acceptor PM for the translocation of GPI-APs released from sources other than PM, such as lipoproteins [53], was tested. Injection of micelle-like complexes, recombinant HDL particles (recHDL), or liposomes reconstituted with AChE (Figure 2e) or CD73 (Figure 2f) or Band-3 (Figure 2e,f; liposomes only) into chips with covalently captured human adipocyte (Figure 2e) or human erythrocyte (Figure 2f) PM caused considerable phase shift increases during prolonged incubation (termination of buffer flow at 1200–4800 s). In contrast, AChE or CD73 reconstituted into TX-100 micelles as well as anchorless AChE or CD73, produced by PI-PLC digestion, exhibited only marginal phase shift increases and/or no increase at all, respectively, compared to control. This argued for the release of GPI-APs from micelle-like complexes, recHDL, and proteoliposomes, and their translocation into acceptor PM at varying efficacy, which was confirmed by subsequent injection (at 5000–5300 s) of anti-AChE (Figure 2e) and anti-CD73 (Figure 2f). Under these conditions, Band-3 was found to resist release from AChE/CD73-Band-3 liposomes (red lines) and/or translocation into human adipocyte (Figure 2e) or erythrocyte (Figure 2f) acceptor PM. At variance, the atypical membrane protein apolipoprotein A-I (Apo-I) was translocated together with AChE and CD73 from AChE/CD73-recHDL, respectively, (blue lines) into both acceptor PM as revealed by anti-Apo-I injection (at 5600–5900 s). This confirmed previous findings [19] about the specificity of intermembrane protein transfer for GPI-APs.

**Figure 3:**
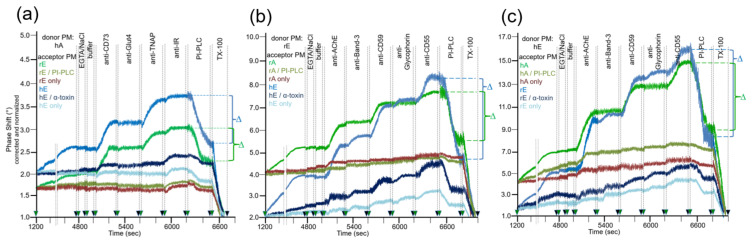
Set-up of chip-based sensing system for transfer of GPI-APs and transmembrane proteins from donor to acceptor PM at various combinations. Human adipocyte (**a**), rat erythrocyte (**b**), and human erythrocyte (**c**) donor PM or washing buffer (acceptor PM only) were injected (at 800–1200 s) into chips with rat erythrocyte (**a**,**c**), human erythrocyte (**a**,**b**), rat adipocyte (**b**), or human adipocyte (**c**) acceptor PM consecutively captured via ionic (Ca^2+^) and covalent bonds as described for Figure 2. The chips were then incubated (1 h, 37 °C) at flow rate 0 (double hatched lines) until 4800 s in the absence or presence of PI-PLC or α-toxin, as indicated. Following injection of EGTA/NaCl and then washing buffer, the protein composition of the acceptor PM was assayed by sequential injection of antibodies against GPI-APs and transmembrane proteins, then of PI-PLC, and finally of TX-100 (0.1%) as indicated. The measured phase shift is given upon correction for unspecific interaction (chips lacking acceptor PM) and normalization for variable capturing efficacy. The differences (∆) between total phase shift upon injection of the last antibody and the phase shift left at the end of injection of PI-PLC are indicated by horizontal hatched lines and brackets as a measure for GPI-AP transfer for each donor–acceptor PM combination. The experiment was repeated two times with similar results.

After having established the conditions for capture of acceptor PM by the TiO_2_ surface of SAW sensing chips and compatible with translocation of GPI-APs upon release from micelle-like complexes, recHDL and proteoliposomes, the possibility of their transfer from donor to acceptor PM was evaluated (Figure 1b). For this, donor PM of various origins were injected into chips with captured acceptor PM of various origin in buffer containing EGTA to avoid Ca^2+^-induced fusion of donor and acceptor PM (Figure 3) and incubated (60 min, 37 °C) by transient termination of the buffer flow (at 1200–4800 s). Following washing of the chip channels with EGTA and NaCl and then buffer to get rid of the donor PM from the microfluidic channels, the captured acceptor PM were assayed for mass loading per se and after sequential injection of antibodies against GPI-APs and transmembrane proteins expressed in the donor PM by real-time measurement of phase shift increases. Incubation of donor PM with acceptor PM at the various combinations (Figure 3, blue and green lines) alone and subsequent injection of anti-CD73 and anti-TNAP, but not anti-Glut4 and anti-IR antibodies (Figure 3a) and anti-AChE, anti-CD59, and anti-CD55, but not anti-Band-3 and anti-Glycophorin antibodies (Figure 3b,c), led to considerable phase shift increases (until 5000 s). Both the donor PM- and antibody-induced phase shift increases were diminished by 65 to 85% in course of subsequent injection of PI-PLC (at 6500–6800 s). This indicated that the corresponding mass loadings onto acceptor PM were mediated by GPI anchorage amenable to cleavage by PI-PLC. The total phase shift increases (i.e., including those induced by capture of the acceptor PM alone) were abrogated by final injection of TX-100 (at 6800–7000 s). This demonstrated dependence of the phase shift increase on the presence of phospholipid layers at the TiO_2_ chip surface and excluded unspecific adsorption of the GPI-APs.

Together, the SAW sensing data are explained best (Figure 1b) by transfer of the GPI-APs CD73 and TNAP from human adipocyte donor PM to rat and human erythrocyte acceptor PM (Figure 3a) and of the GPI-APs AChE, CD59, and CD55 from rat (Figure 3b) and human erythrocyte donor PM (Figure 3c) to rat and human adipocyte and erythrocyte acceptor PM. The specificity of the transfer for GPI-APs was demonstrated (Figure 3a–c) by (i) failure of typical transmembrane proteins to elicit corresponding phase shift increases and (ii) complete blockade and considerable reduction, respectively, of phase shift increase in the presence of PI-PLC or α-toxin during incubation of donor and acceptor PM (at 1200–4800 s). (ii) was most likely caused by lipolytic cleavage of the GPI-APs to be transferred and inhibition of transfer due to binding of α-toxin to the GPI core glycan, respectively [54,55].

The omission of donor PM during the incubation revealed the endogenous expression of the relevant GPI-APs and transmembrane proteins at the acceptor PM determined by their differential species- and tissue-specific expression as well as the differential species-specific cross-reactivity of the antibodies used (Table 1). Rat and human erythrocyte PM harbored a low amount of IR (Figure 3a; at 5900–6200 s), rat adipocyte PM of AChE (Figure 3b,c; at 5000–5300 s). Human and rat erythrocyte PM expressed low amounts of AChE, Band-3, CD59, Glycophorin, and CD55 (Figure 3b,c; at 5000–6500 s). For transmembrane proteins, the antibody-induced phase shift increases were very similar for incubations of acceptor PM only and of donor with acceptor PM, confirming failure of their transfer. For GPI-APs, the increases were considerably higher for incubations of donor with acceptor PM compared to incubation of acceptor PM only, which was compatible with their transfer from donor to acceptor PM. With regard to GPI-APs, the unequivocal demonstration of their transfer from donor to acceptor PM for the six combinations assayed was enabled by differential species-/tissue-specific GPI-AP expression and/or differential species-specific antibody reactivity (Table 1). The difference Δ between the maximal phase shift increase at 6500 s (in course of sequential injection of the donor PM and the set of antibodies as indicated) and the phase shift increase left upon injection of PI-PLC at 6800 s (Δ phase shift) was calculated for each combination of donor and acceptor PM (see Figure 3) and used as a measure for the transfer efficacy in the following experiments.

**Table 1:** Synopsis of the various combinations of donor and acceptor PM including the experimental basis enabling analysis of the transfer of GPI-APs, and the comparison of the relative transfer efficacy. Relative transfer efficacy is derived from Figure 4a (with 400 µL of donor PM injected) and categorized as follows: +, 0.5–1.0 Δ phase shift; +++, 2.0–3.0; ++++, 3.0–4.0; +++++, 5.0–6.0; ++++++, 6.0–7.0.

Combination			Experimental Basis		Relative Transfer Efficacy
Donor PM	Acceptor PM	Abbreviation	Differential Species-/Tissue-Specific GPI-AP Expression	Differential Species-Specific Antibody Reactivity	
human adipocyte	human erythrocyte	hA → hE	yes	yes	+
rat erythrocyte	human erythrocyte	rE → hE	no	yes	++++
human erythrocyte	human adipocyte	hE → hA	yes	yes	+++++
human erythrocyte	rat erythrocyte	hE → rE	no	yes	++++++
rat adipocyte	rat erythrocyte	rA → rE	yes	no	+
rat erythrocyte	rat adipocyte	rE → rA	yes	no	+++

**Figure 4:**
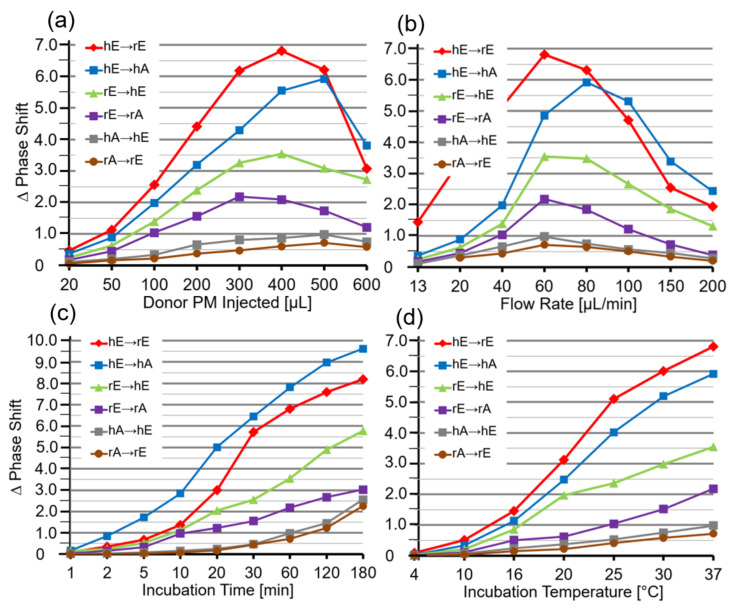
Optimization of chip-based sensing system for transfer of GPI-APs and membrane proteins from donor to acceptor PM. Dependence of transfer efficacy on the amount of donor PM (**a**), flow rate during donor PM injection (**b**), length of transfer period (**c**), temperature during transfer (**d**). The experiment was performed as described for Figure 3 with injection of donor PM at 800 s, and start of incubation of the donor–acceptor PM combinations indicated at 1200 s in the absence or presence of PI-PLC (in the absence of α-toxin) (**a**) with increasing volumes of the donor PM at a flow rate of 60 μL/min for 60 min at 37 °C, (**b**) at increasing flow rates with 400 μL of donor PM for 60 min at 37 °C, (**c**) for increasing incubation periods with 400 μL of donor PM at flow rate 0 at 37 °C and (**d**) at increasing temperatures with 400 μL of donor PM at a flow rate of 60 μL/min for 60 min. ∆ phase shifts as measure for GPI-AP transfer are calculated as described for Figure 3. The experiments were repeated two times with similar results. Mean values are given for each donor–acceptor PM combination.

Next, critical parameters for the efficacy of the transfer of GPI-APs using this experimental set-up were investigated, such as the amount of donor PM injected into the chip and then incubated with the acceptor PM (Figure 4a), the flow rate during the initial injection of the donor PM (Figure 4b), the time of incubation of donor and acceptor PM at flow rate 0 (Figure 4c), and the incubation temperature (Figure 4d). Maximal transfer efficacy was observed at 300–500 µL of PM (corresponding to 60–100 µg of protein) and 60–80 µL/min flow rate, with approximate linear correlation with incubation time between 10 and 60 min and temperature between 20 and 37 °C. Only minor differences were found between the six donor–acceptor PM combinations (Figure 4). Therefore, injection of 400 µL of PM at 60 µL/min flow rate and subsequent incubation (60 min, 30 °C) were used for the following experiments. Under these optimal conditions, the transfer of GPI-APs from donor to acceptor PM was most efficient for the combinations hE → rE and hE → hA and least for hA → hE and rA → rE (Table 1).

The apparent specificity of the GPI-AP transfer, as reflected in the exclusion of transmembrane proteins from expression at the acceptor PM (see Figure 3), provided a first hint that the experimental set-up, in particular the absence of Ca^2+^ during injection and incubation of the donor and acceptor PM, did not support vesicle fusion. For clarification as to whether fusion of donor and acceptor PM can be provoked at the chip surface under special conditions and monitored as SAW phase shift, donor PM were injected together with Ca^2+^, known to trigger phospholipid bilayer fusion in vitro [56,57], into chips with covalently captured acceptor PM (Figure 1d, left panel). Following incubation, subsequent removal of Ca^2+^, and then washing with NaCl (Figure 1d, middle panel), the chip TiO_2_ surface was assayed for the presence of GPI-APs and transmembrane proteins by successive injection of corresponding antibodies (Figure 1d, right panel).

**Figure 5:**
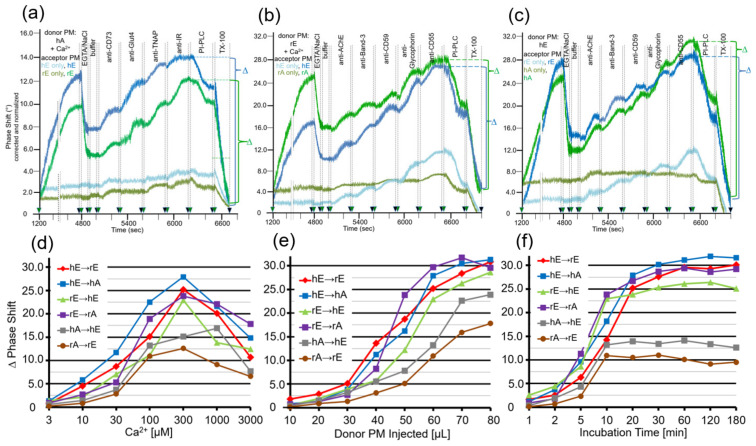
Ca^2+^-dependent fusion of donor and acceptor PM harboring GPI-APs and transmembrane proteins at various combinations (**a**–**c**) and its dependence on the amount of donor PM (**d**), length of the incubation period (**e**) and concentration of Ca^2+^ (**f**). The experiment was performed as described for Figure 3 with injection at 800–1200 s of 85 μL (**a**–**d**,**f**) or increasing volumes (**e**) of donor PM at a flow rate of 13 μL/min and subsequent incubation (37 °C) of the donor–acceptor PM combinations or acceptor PM only as indicated (in the absence of PI-PLC and α-toxin) in the presence of 100 μM Ca^2+^ (**a**–**c**,**e**,**f**) or increasing concentrations (**d**) for 60 min (1200–4800 s, (**a**–**e**)) or increasing periods of time (**f**). ∆ phase shifts as measure for GPI-AP transfer are calculated as described for Figure 3. The experiments were repeated two times with similar results. Mean values are given for each donor–acceptor PM combination (**d**–**f**).

The covalently captured human/rat erythrocyte and adipocyte acceptor PM were found to be constituted of small amounts of CD73, TNAP, IR (Figure 5a; erythrocyte), and AChE, Band-3, CD59, Glycophorin, CD55 (Figure 5b,c; adipocyte), and of small amounts of AChE, CD59, CD55 (Figure 5b,c; adipocyte) as measured upon omission of donor PM injection (h/rE/A only, light green and blue lines). Injection of human adipocyte (Figure 5a), rat erythrocyte (Figure 5b), or human erythrocyte (Figure 5c) donor PM together with Ca^2+^ (at 1200–4800 s) led to drastic increases in phase shift for each of the acceptor PM, about half of which resisted subsequent washing with EGTA/NaCl (at 4800–4900 s). Strikingly, injection of antibodies against both GPI-APs and transmembrane proteins (at 5000–6200 s) led to pronounced phase shift increases (Figure 5a–c; dark green and blue lines). These findings were explained best by Ca^2+^-induced fusion of donor and acceptor PM vesicles. The 25–35% phase shift lowering in response to PI-PLC injection (at 6200–6500 s) confirmed that the major portion of the donor PM-dependent mass loading is due to transmembrane proteins and a minor one to GPI-APs. Phase shift increases by both transmembrane proteins and GPI-APs were completely abrogated by injection of TX-100, which apparently caused disintegration of the fused donor–acceptor PM vesicles (Figure 5a–c).

Thus, fusion of donor and acceptor PM at the chip surface could be achieved for each combination (Figure 1d, right panel), but strictly depended on the presence of Ca^2+^ with optimum at 300 µM (Figure 5d). This, together with the considerable deviations in the amount of donor PM (Figure 5e) and incubation time (Figure 5f) leading to maximal phase shift increases (60–80 µL vs. 300–500 µL; 20–30 min vs. 60–180 min) with incubations of donor and acceptor PM in the presence (Figure 5) vs. absence (Figure 4) of Ca^2+^, strongly argued for fusion of PM vesicles under the former and transfer of GPI-APs under the latter conditions. Both was monitored and distinguished from one another by chip-based SAW sensing.

### 3.2. Transfer of Full-Length GPI-APs between Rat PM at Various Combinations Depends on the Metabolic State of the Rats

Previous studies have demonstrated that full-length GPI-APs, i.e., those harboring the complete GPI anchor with the fatty acid moieties remaining attached, can be released from the surface of tissue and blood cells into the blood stream of rats and humans [58,59,60]. Interestingly, the release was reported to be increased in obese and diabetic compared to normal rats and humans, albeit lower serum concentrations of full-length GPI-APs were measured for the former vs. the latter [30]. This inverse relationship between the rate of release of GPI-APs and their steady state concentration in serum was attributed to enhanced degradation of the released GPI-APs by lipolytic cleavage of their GPI anchor through serum GPI-specific phospholipase D (GPLD1). Its activity and amount were found to be elevated in obese and diabetic rats and humans [31]. Nevertheless, the measured upregulation of GPLD1 did not exclude the possibility that a portion of full-length GPI-APs released from donor tissue or blood cells manage to escape cleavage by GPLD1. Consequently, those may find their path to acceptor cells in the same or a neighboring tissue depot via a paracrine route or to distinct tissue or blood acceptor cells via an endocrine route, and finally become translocated in the outer phospholipid bilayer of their PM.

**Figure 6:**
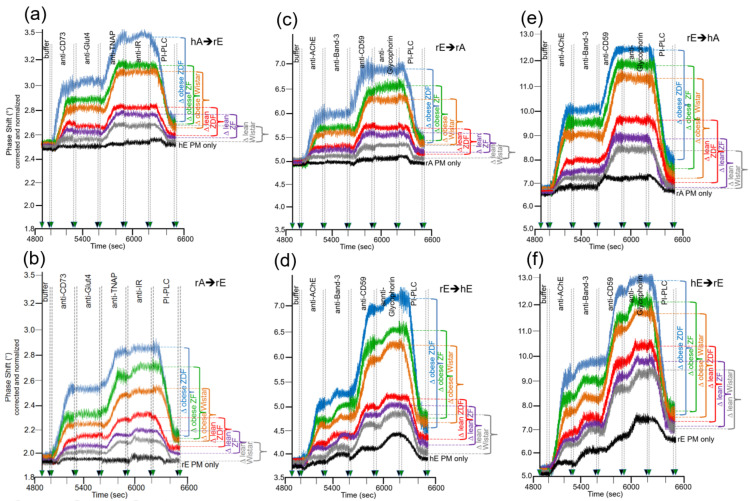
Chip-based sensing system for the transfer of full-length GPI-APs from donor to acceptor PM at various combinations of the six rat groups. (**a**–**f**) The experiment was performed as described for Figure 3 with injection of 400 μL of donor PM (800–1200 s) at a flow rate of 60 μL/min and subsequent incubation (until 4800 s, 60 min, 37 °C) of the donor–acceptor PM combinations or acceptor PM only as indicated (donor PM → acceptor PM). At variance with Figure 3, injection of anti-CD55 antibody was omitted for the combinations with donor erythrocytes (**c**–**f**). The rat (r) donor and acceptor PM were derived from adipocytes (A) and erythrocytes (E) which had been prepared from the six rat groups. Phase shifts are shown only after termination of the transfer period/start of buffer injection (4800 s) and termination of PI-PLC injection (6500 s). ∆ phase shifts as measure for GPI-AP transfer are calculated as described for Figure 3.

Next, the sensing system for transfer of full-length GPI-APs from donor to acceptor PM under conditions which do not support vesicle fusion was used to investigate whether there are differences in the transfer of GPI-APs dependent on the metabolic state of the rats which serve as source for the donor to acceptor PM. Putative correlations between transfer efficacy and metabolic state would argue for relevance in vivo of GPI-AP transfer. For this, PM were prepared from primary epididymal adipocytes and erythrocytes from six groups of rats, which differ in genotype, feeding state, and metabolic phenotype (Table 2) and were used as donors as well as acceptors for GPI-APs at various combinations. In addition, PM from human erythrocytes were used as “neutral” donors and acceptors, respectively, to check for the metabolic relevance of donor vs. acceptor PM. The acceptor PM were assayed for the presence of transferred GPI-APs by mass loading onto the chip of antibodies against GPI-APs and transmembrane proteins (Figure 6).

Considerable differences in transfer of GPI-APs (at 5000–6200 s) were monitored between the six rat groups with identical ranking for the six donor–acceptor PM combinations (Figure 6a–f). In all cases, transfer efficacy was considerably higher than that measured during omission of injection of the corresponding donor PM (PM only). This confirmed the species- and tissue-specific expression and detection of the GPI-APs and transmembrane proteins studied. In agreement, the phase shift increases caused by the acceptor PM only were more pronounced for erythrocytes “homologously” assayed for the transfer of erythrocyte proteins (Figure 6d,f) compared to erythrocyte or adipocyte PM “heterologously” assayed for adipocyte and erythrocyte proteins, respectively (Figure 6a–c,e). This confirmed the species and tissue specificity of the antibodies used.

Transfer of adipocyte CD73 and TNAP (Figure 6a,b), as well as erythrocyte AChE and CD59 (Figure 6c–f), were highest for obese ZDF rats exhibiting elevated fasting blood glucose (hyperglyemia) and elevated fasting plasma insulin (hyperinsulinemia) levels, followed by obese ZF rats with normal fasting blood glucose (normoglycemia) and hyperinsulinemia and obese normoglycemic Wistar rats with mild hyperinsulinemia. Lean normoglycemic ZDF with mild hyperinsulinemia and lean normoglycemic ZF rats with normal fasting plasma insulin (normoinsulinemia) displayed intermediary GPI-AP transfer, which was slightly above that of lean normoglycemic normoinsulinemic Wistar rats. Importantly, in each donor–acceptor PM combination, no or only very minor transfer of adipocyte Glut4 and IR (Figure 6a,b), as well as erythrocyte Band-3 and Glycophorin (Figure 6c–f), was detectable. Again, this demonstrated the specificity of transfer for GPI-APs.

**Figure 7:**
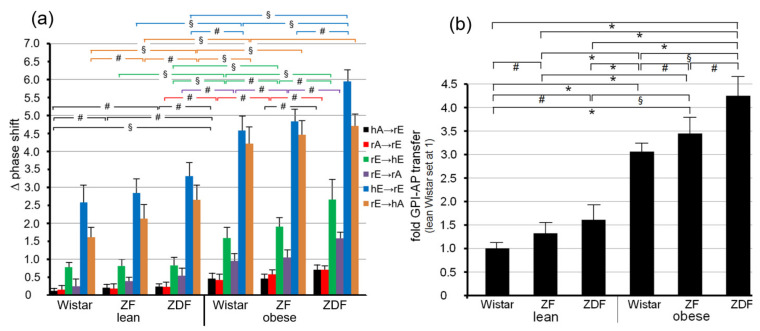
Comparative quantitative evaluation of the six rat groups for transfer of full-length GPI-APs from donor to acceptor PM for the various combinations (**a**) and the calculated means thereof (**b**). The experiment was performed as described for Figure 6 with measurements in quadruplicate (with distinct chips each) for each donor–acceptor PM combination. (**a**) ∆ phase shifts as measure for GPI-AP-induced increases in phase shift are calculated as described for Figure 6 and given as means ± SD for each combination with statistical significance (^§^ *p* ≤ 0.02, ^#^ *p* ≤ 0.05; only between rat groups displaying relatively small differences for reasons of clarity). (**b**) Fold GPI-AP transfer was calculated relative to control (acceptor PM only, Figure 6) for each of the six rat groups upon calculation of the means for the donor–acceptor PM combinations for each rat group and normalization of lean Wistar rats (set at 1) as means ± SD with statistical significance (* *p* ≤ 0.01, ^§^ *p* ≤ 0.02, ^#^ *p* ≤ 0.05 between all rat groups).

Quantitative evaluation of the transfer efficacy for total GPI-APs (Figure 7a) revealed prominent differences (at 5000–6200 s) between the various donor–acceptor PM combinations with identical ranking for each rat group with decreasing efficacy in that order: hE → rE > r/hE → hA > rE → hE > rE → rA > rA → rE = hA → h/rE. Apparently, the transfer efficacy was determined by both donor and acceptor PM, since a given donor or acceptor PM led to different transfer efficacy when assayed with different acceptor or donor PM, respectively. Apparently, the release of GPI-APs from donor PM as well as their translocation into acceptor PM were critical for transfer of GPI-APs between PM. Both the differential transfer efficacy of GPI-APs as assayed for the various donor–acceptor PM combinations in vitro (Figure 5) and their varying potency to accomplish differentiation between the rats of the six different metabolic phenotypes (Figure 7a) may be explained by subtle differences in the biophysical and biochemical characteristics of the PM, such as stiffness, viscoelasticity, and fluidity, which determine the release and/or translocation of GPI-APs and thus their transfer between tissue and blood cells in vivo.

Consequently, maximal differentiation power was obtained by summation of the phase shift differences measured for all six donor–acceptor PM combinations for each of the six rat groups and calculation of the fold GPI-AP transfer (Figure 7b). This resulted in significant differences between each of the six rat groups in that ranking order of increasing transfer efficacy: lean Wistar < ZF < ZDF < obese Wistar < ZF < ZDF.

### 3.3. Transfer of Full-Length GPI-APs between Rat PM at Various Combinations Is Impaired by Serum Proteins, among Them GPLD1

**Figure 8:**
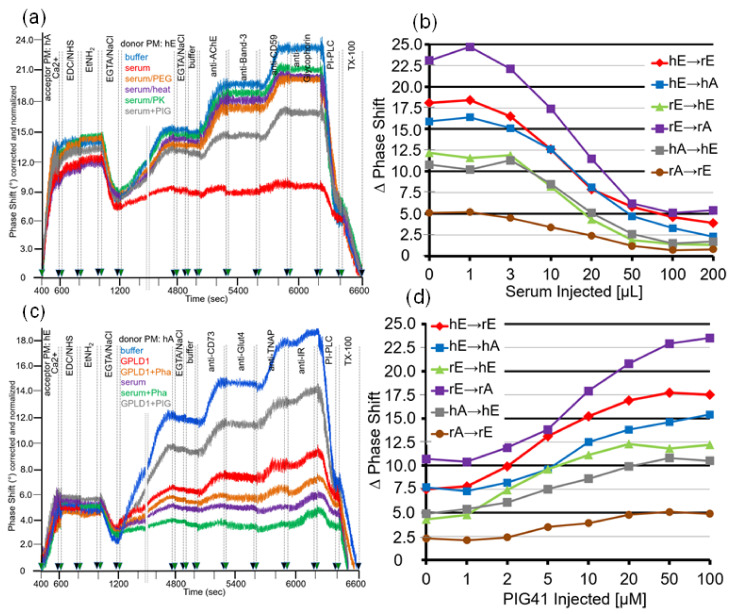
Effect of serum proteins and PIG on the transfer of full-length GPI-APs from donor to acceptor PM at various combinations. 400 μL of human erythrocyte (**a**) or adipocyte (**c**) donor PM were injected at 1200 s and at a flow rate of 60 μL/min into chips with human adipocyte (**a**) and erythrocyte (**c**), respectively, acceptor PM captured by ionic (Ca^2+^) and covalent bonds (EDC/NHS). (**a**,**c**) After blockade with EtNH_2_ and washing with EGTA/NaCl as described for Figure 2, 100 μL of washing buffer or serum from obese rats (diluted 5-fold with buffer), which had been treated with PEG6000, heat, proteinase K (PK), or supplemented with GPLD1 (0.4 units) alone or together with 30 μM PIG41 or 100 µM Pha, were injected as indicated. Thereafter, the chips were incubated until 4800 s at 37 °C at flow rate 0 (double hatched lines). Following injection of EGTA/NaCl and then washing buffer, the protein composition of the acceptor PM was assayed by consecutive injection of antibodies against GPI-APs and transmembrane proteins, then of PI-PLC, and finally of TX-100 as indicated. The phase shift measured is given upon correction for unspecific interaction (no acceptor PM) and normalization for variable capturing efficacy. The experiment was repeated once with similar results. (**b**) Increasing volumes of rat serum (diluted 5-fold) or buffer or (**d**) 20 μL of rat serum, together with increasing concentrations of PIG41 or buffer, were injected. ∆ phase shifts as measure for GPI-AP transfer are calculated as described for Figure 3. The experiments were repeated two times with similar results. Mean values are given for each donor–acceptor PM combination.

For mimicking of the conditions for the transfer of GPI-APs in vivo, in particular with regard to the milieu surrounding the donor and acceptor tissues and blood cells, by the SAW chip-based sensing system, the buffer present during the incubation of donor and acceptor PM (at 1200–4800 s) was supplemented with serum (Figure 1c). As expected, two-step ionic (at 400–600 s) and then covalent capture (at 600–800 s) of human adipocyte acceptor PM followed by capping of reactive groups (at 800–1000 s) and then removal of Ca^2+^ (at 1000–1200 s) resulted in pronounced mass loading onto the chip surface (Figure 8a; see Figure 2 for explanation). Injection of diluted serum from lean Wistar rats together with human erythrocyte donor PM (at 1200–4800 s) led to considerably diminished transfer of AChE and CD59 (red line) compared to the absence of serum (blue line). The use of serum depleted of proteins by PEG precipitation (orange line) or heat treatment (pink line) or proteinase K digestion (green line) or of serum supplemented with synthetic phosphoinositolglycan41 (PIG, brown line), which resembles the structure of the GPI anchor core glycan [61], impaired the serum-induced reduction in GPI-AP transfer at varying degrees. Apparently, rat serum contains proteins which interfere with transfer of GPI-APs, in part by interaction with the core glycan of their GPI anchor, which is competed for by synthetic PIG. The specificity of serum inhibition of transfer was confirmed by the missing effect on the transmembrane proteins, Band-3 and Glycophorin (Figure 8a).

Inhibition of GPI-AP transfer by serum from lean Wistar rats held true for each of the six donor–acceptor PM combinations and was dependent on the volume injected with maximal effect at 50–100 µL (Figure 8b).

As a putative candidate for a serum protein inhibiting GPI-AP transfer, GPLD1 was considered based on its interaction with and cleavage of the GPI anchor of GPI-APs [40,62,63]. Consequently, the experiment was repeated with recombinant GPLD1 instead of total serum (Figure 8c). In fact, injection of GPLD1 together with human adipocyte donor PM into chips with covalently captured human erythrocyte acceptor PM led to considerable lowering of transfer (at 1200–4800 s, red line) compared to buffer (blue line) which, however, was less pronounced than that provoked by total serum proteins (pink line). Interestingly, ortho-phenanthroline (Pha), which inhibits GPLD1 by chelating of divalent cations [62,63], further enhanced inhibition of GPI-AP transfer by both GPLD1 (orange line) and total serum proteins (green line). Strikingly, PIG41 partially relieved downregulation of transfer by GPLD1 (Figure 8c; brown line).

Restoration of GPLD1-blocked GPI-AP transfer by PIG41 was observed with each of the six donor–acceptor PM combinations and found to depend on the concentration with half-maximal effect at 7–10 µM (Figure 8d). Taken together, these findings strongly suggested that (i) GPLD1 represents one of the serum proteins interfering with transfer of GPI-APs and (ii) its inhibitory potency is due to interaction with the core glycan rather than lipolytic cleavage of the GPI anchor of GPI-APs (Figure 1c).

### 3.4. Transfer of GPI-APs between Rat PM Depends on the Assembly of GPI Anchors, Phospholipids, and Cholesterol in Non-Vesicular Structures

Previous studies have demonstrated that full-length GPI-APs reconstituted in vitro with phospholipids and cholesterol at certain composition and ratio into micelle-like complexes [32] become translocated into the outer leaflet of isolated or cellular PM under maintenance of their biological function [33]. Similar complexes have recently been identified in serum of rats and humans and their levels were found to be reduced in diabetic and obese rats and humans compared to normal ones [31]. Furthermore, in the present investigation, the translocation of AChE from micelle-like complexes into human adipocyte and erythrocyte acceptor PM was analyzed using the novel chip-based SAW sensing system. Furthermore, this system enabled the discrimination between transfer of GPI-APs from donor to acceptor PM and fusion of donor and acceptor PM (see Figure 5). Taking the available data together, it was tempting to speculate that the transfer of full-length GPI-APs from donor to acceptor PM is mediated by micelle-like complexes rather than membrane structures.

**Figure 9:**
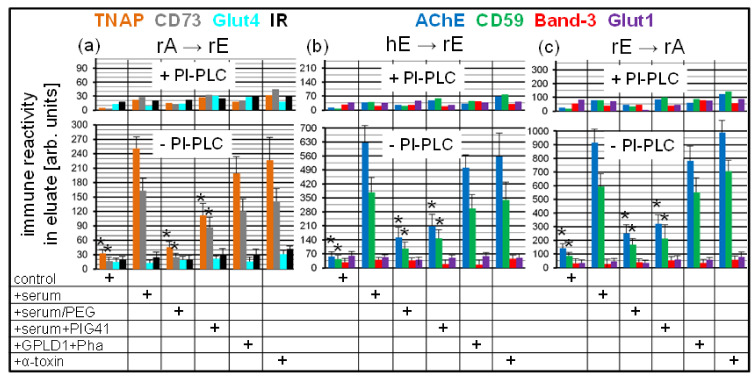
Analysis of the chip eluate for membrane proteins released from the donor PM upon blockade of transfer of full-length GPI-APs to acceptor PM at various combinations. Rat adipocyte (**a**), human erythrocyte (**b**), and rat erythrocyte (**c**) donor PM were injected at 1200 s and at a flow rate of 60 μL/min into chips with rat erythrocyte (**a**,**b**) or rat adipocyte (**c**) acceptor PM, respectively, consecutively captured via ionic (Ca^2+^) and covalent bonds (EDC/NHS), blocked with EtNH_2_ and then washed with EGTA/NaCl as described for Figure 8. Thereafter, 100 μL of washing buffer (control) or serum from obese rats (diluted five-fold with buffer), which had been treated with PEG6000 or left untreated, alone or together with 30 μM PIG41 or GPLD1 (0.4 units) together with 100 µM Pha or α-toxin (10 µg/mL) were injected as indicated. Thereafter, the chips were incubated until 4800 s at 37 °C at flow rate 0. Following injection of 100 µL of EGTA/NaCl at a flow rate of 60 µL/min and then of 400 µL of washing buffer at the same flow rate, the eluate from the chip channels was collected from 4900 to 5300 s and then centrifuged (100,000× *g*, 1 h, 4 °C). The supernatants were removed, and halves incubated (2 h, 30 °C) in the absence (lower panels) or presence (upper panels) of PI-PLC as described in the Methods section. Following TX-114 partitioning of the incubation mixtures, the detergent-enriched phases were analyzed for the presence of GPI-APs and transmembrane proteins as indicated by dot blotting with antibodies against TNAP, CD73, AChE, CD59, Glut4, IR, Band-3 and Glut1 as described in the Methods section. “+” below the bars denotes the injection of no serum (control), +serum, PEG-treated serum (+serum/PEG), serum together with PIG41 (+serum+PIG41), GPLD1 together with Pha (+GPLD1+Pha) and α-toxin (+α-toxin), respectively. The immune reactivities (arb. units) are given as means ± SD (four incubations and elutions each with distinct chips) for each eluted protein (with dot blotting in triplicate) upon normalization by subtraction of unspecific signals generated in the absence of antibody (* *p* ≤ 0.01 vs. untreated serum alone).

To test for the possibility that micelle-like GPI-AP complexes are generated in the chip channels in course of transfer of GPI-APs, donor PM were injected into chips with covalently captured acceptor PM at various combinations and incubated (at 1200–4800 s) in the absence (control) or presence of un- or pretreated serum proteins or α-toxin. Then, the microfluidic chip channels were eluted, and the collected eluates were centrifuged to get rid of any membrane structures including the donor PM. The supernatants were digested with PI-PLC or left untreated for discrimination between structures harboring full-length GPI-APs and GPI-APs lipolytically released from the donor PM. After TX-114 partitioning, the detergent-enriched phases were analyzed for the presence of full-length GPI-APs and transmembrane proteins by dot blotting with corresponding antibodies (Figure 9).

Quantitative evaluation of the immune reactivity of the dots revealed considerable amounts of the GPI-APs, TNAP and CD73, in the undigested (-PI-PLC) chip eluates generated by the rA → rE (Figure 9a), and AChE and CD59 by the hE → rE (Figure 9b) and rE → rA (Figure 9c) combinations in the presence of total serum proteins or blocked (by Pha) GPLD1 or α-toxin, i.e., under conditions which have been shown to interfere with the transfer of GPI-APs (see Figure 8). For each combination, the amounts of eluted GPI-APs in the detergent-enriched phase were significantly reduced upon omission of serum proteins (control) or use of serum depleted of proteins by PEG precipitation or use of serum in combination with PIG41. The almost complete removal of GPI-AP immune reactivities from the detergent-enriched phase upon digestion with PI-PLC for all combinations demonstrated the generation of full-length GPI-APs equipped with the complete GPI anchor in the chip channels during transfer from donor to acceptor PM (Figure 9a–c). Only minute amounts of immune-reactive transmembrane proteins Glut4, IR, Band-3, and Glut1, irrespective of the donor–acceptor PM combination, were detectable in the (undigested or digested) chip eluates.

Together, the data are explained best by specific assembly of full-length GPI-APs into non-membrane structures in the chips in course of blockade of transfer by serum proteins, such as GPLD1, or α-toxin. The blockade was presumably caused by their binding to the GPI anchors and apparently prerequisite for the accumulation of full-length GPI-APs in the chip channels at amounts, which were detected by dot blotting. It is tempting to speculate that full-length GPI-APs in non-membrane structures (which cannot be spun down by centrifugation at 100,000× *g*) are also generated in the course of (unblocked) transfer following release from the donor PM, however for a short period only, due to rapid translocation into acceptor PM.

**Figure 10:**
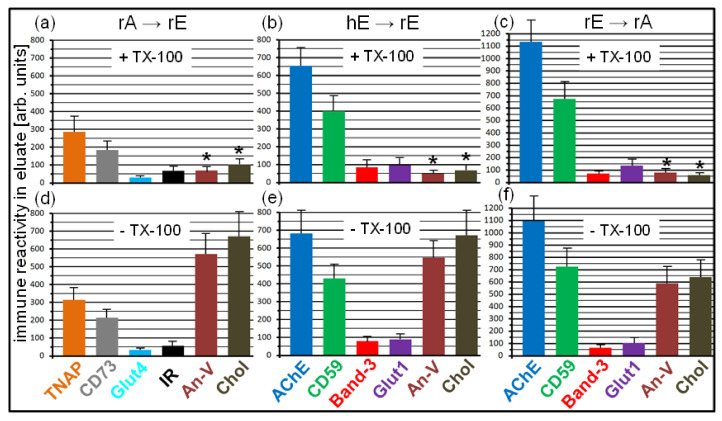
Analysis of the chip eluate for micelle-like GPI-AP complexes released from the donor PM upon blocked transfer of full-length GPI-APs to acceptor PM at various combinations. Rat adipocyte (**a**,**d**), human erythrocyte (**b**,**e**), and rat erythrocyte (**c**,**f**) donor PM were injected into chips with rat erythrocyte (**a**,**b**,**d**,**e**) or rat adipocyte (**c**,**f**) acceptor PM as described for Figure 9. Following injection of 100 μL of serum from obese ZDF rats (diluted five-fold with buffer) together with 100 µM Pha, the chips were incubated until 4800 s at 37 °C at flow rate 0. Following injection of 100 µL of EGTA/NaCl at a flow rate of 60 µL/min and then of 400 µL of washing buffer at the same flow rate, the eluate from the chip channels was collected from 4900 to 5300 s and then centrifuged (100,000× *g*, 1 h, 4 °C). The supernatants were removed, and halves incubated in the absence (**d**–**f**) or presence (**a**–**c**) of TX-100 (0.1%) for 1 h at 30 °C and then with α-toxin coupled to Sepharose beads as described in the Methods section for 20 h at 4 °C (head-over rotation). The mixtures were centrifuged (10,000× *g*, 5 min, 4 °C). The pellets were washed three times by suspending in washing buffer and recentrifugation. The final pellets were suspended in the same volume of two-fold Laemmli sample buffer and heated (5 min, 65 °C). Following centrifugation (10,000× *g*, 5 min, 25 °C), the supernatants were assayed for the presence of GPI-APs and transmembrane proteins by dot blotting with antibodies against TNAP, CD73, AChE, CD59, Glut4, Glut1, Band-3 and Annexin-V as described in the Methods section. Portions of the washed and Laemmli-extracted α-toxin Sepharose beads were determined for cholesterol. The immune reactivities and cholesterol amounts (arb. units) are given as means ± SD (four distinct transfer incubations and chip elutions each) with dot blotting in triplicate each upon normalization by subtraction of unspecific signals generated in the absence of antibody and Sepharose beads, respectively (* *p* ≤ 0.01 vs. incubation in the absence of TX-100).

Next, the nature of the non-membrane structures apparently harboring the full-length GPI-APs, was characterized (Figure 10). For this, donor PM were injected into TiO_2_ chips with covalently captured acceptor PM at various combinations and then incubated under conditions of maximal blockade of GPI-AP transfer (see Figure 8c), i.e., in the presence of serum from obese ZDF rats and Pha. Then, the eluates of the chip channels were collected and centrifuged to get rid of any membrane structures, including the donor PM. Portions of the supernatants were treated with TX-100 or left untreated and thereafter incubated with α-toxin coupled to Sepharose beads. After centrifugation, the collected beads were extracted with SDS (Laemmli buffer) and then analyzed by dot blotting with antibodies against GPI-APs, transmembrane proteins, and peripheral membrane proteins (annexin-V). In addition, portions of the SDS extracts were determined for cholesterol contents. Both annexin-V and cholesterol have recently been demonstrated to be constituents of micelle-like GPI-AP complexes in rat und human serum [33].

Quantitative evaluation of the immune reactivity of the dots revealed considerable amounts of the GPI-APs TNAP and CD73 or AChE and CD59 in the TX-100-treated (upper panels) as well as untreated (lower panels) chip eluates generated by the rA → rE (Figure 10a,d) and hE → rE (Figure 10b,e) as well as rE → rA (Figure 10c,f) combinations, respectively, in the presence of total serum proteins, including blocked GPLD1. In contrast, only minute amounts of the transmembrane proteins Glut4, IR, Band-3, and Glut1 were detectable, irrespective of the combination and treatment of the eluate with or without TX-100. Strikingly, annexin-V and cholesterol were detected in untreated eluates of each combination at considerable amounts (Figure 10d–f) but were significantly diminished upon treatment with TX-100 (Figure 10a–c). These data strongly suggested that in course of blockade of GPI-AP transfer, full-length GPI-APs accumulate in the chip channels which are embedded together with the phospholipid-binding protein annexin-V and cholesterol in detergent-sensitive non-membrane structures. It is tempting to speculate that those structures are similar to micelle-like GPI-AP complexes constituted by phospholipids, lysophospholipids, and cholesterol at certain ratios as previously described [30,33] and mediate the transfer of GPI-APs from donor to acceptor PM in the chip in the absence of serum proteins.

### 3.5. Control of Transfer of GPI-APs between Rat PM at Various Combinations by Serum Proteins Depends on the Metabolic State of the Rats

**Figure 11:**
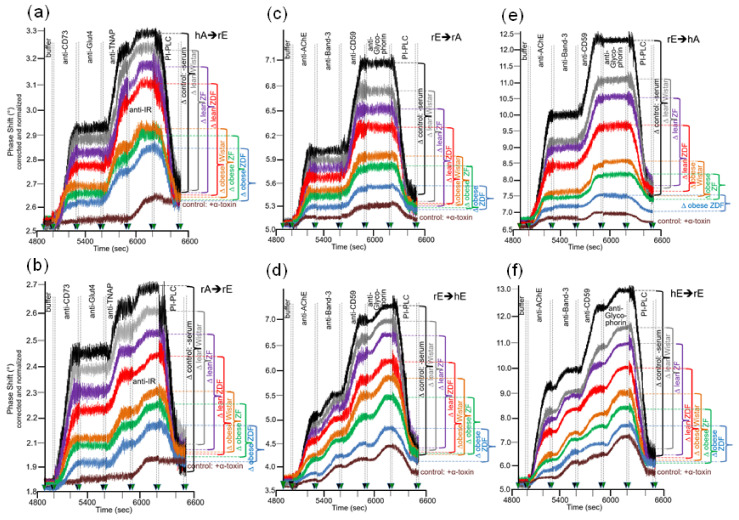
Determination of the effect of serum proteins from the six rat groups on the transfer of full-length GPI-APs from donor to acceptor PM at various combinations. The experiment was performed as described for Figure 6 with injection of 400 μL of donor PM (800–1200 s) at a flow rate of 60 μL/min and subsequent incubation (until 4800 s, 60 min, 37 °C) of the donor–acceptor PM combinations ((**a**), hA → rE; (**b**), rA → rE; (**c**), rE → rA; (**d**), rE → hE; (**e**), rE → hA; (**f**), hE → rE) in the absence (control: -serum) or presence of 100 μL serum (diluted five-fold) from the six rat groups or in the presence of 10 μg/mL α-toxin (control: +α-toxin) as indicated (donor PM → acceptor PM). At variance with Figure 6, the rat (r) donor and acceptor PM were derived from adipocytes (A) and erythrocytes (E) which had been isolated from obese ZDF rats. Phase shifts are shown only between start of buffer injection (4800 s) and termination of PI-PLC injection (6600 s). ∆ phase shifts as measure for GPI-AP transfer are calculated as described for Figure 3.

**Figure 12:**
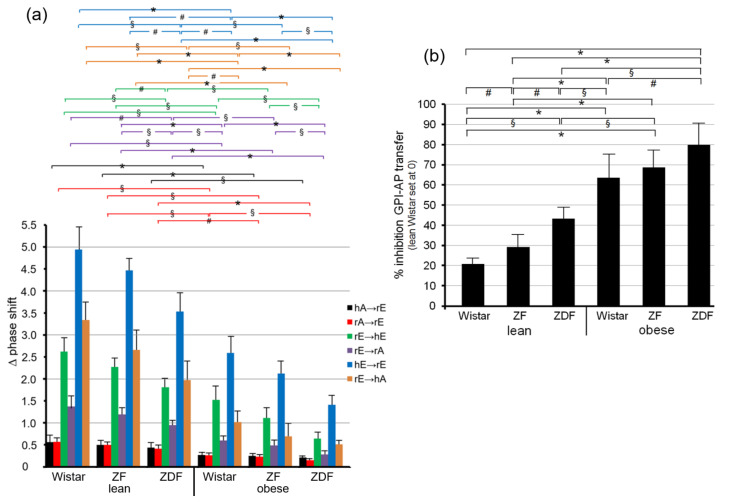
Comparative quantitative evaluation for the six rat groups of the inhibition of transfer of full-length GPI-APs from donor to acceptor PM at the various combinations (**a**) and the calculated means thereof (**b**). The experiment was performed as described for Figure 11 with measurements repeated six times for each donor–acceptor PM combination (different incubations with distinct chips each). (**a**) ∆ phase shifts as measure for GPI-AP-induced increases in phase shift are calculated as described for Figure 7 and given as means ± SD for each combination with statistical significance (* *p* ≤ 0.01, ^§^ *p* ≤ 0.02, ^#^ *p* ≤ 0.05) indicated only for rat groups displaying relatively small differences (for reasons of clarity). (**b**) % inhibition of GPI-AP transfer was calculated relative to control (-serum, Figure 11) for each of the six donor–acceptor PM combinations and each of the six rat groups upon normalization of lean Wistar rats (set at 0) as means ± SD with statistical significance (* *p* ≤ 0.01, ^§^ *p* ≤ 0.02, ^#^ *p* ≤ 0.05) indicated between all rat groups.

The above observation (see Figure 8) demonstrated that rat serum proteins, among them GPLD1, interfere with the transfer of GPI-APs from donor to acceptor PM. Previous findings revealed differential interaction of GPI-APs with serum proteins from rats of varying metabolic phenotype [32]. Together, this raised the possibility of inhibition of GPI-AP transfer by serum proteins in relation to the metabolic state of the rats, which was tested by the final set of experiments (Figure 11 and Figure 12).

Lowering of GPI-AP transfer by serum proteins was monitored for each of the six rat groups using the above six donor–acceptor PM combinations (Figure 11). Transfer of adipocyte CD73 and TNAP (Figure 11a,b), as well as erythrocyte AChE and CD59 (Figure 11c–f), were lowest for obese ZDF rats. This presumably reflected the most pronounced blockade of GPI-AP transfer, which was almost as potent as that provoked by α-toxin (control for maximal inhibition). For obese ZF and Wistar rats and lean ZDF and ZF rats, intermediary levels of GPI-AP transfer in this ranking order of declining potency were measured, compatible with its intermediary blockade. Lean Wistar rats displayed highest transfer and thus the least potent blockade. Importantly, for each of the six donor–acceptor PM combinations incubated with serum from each of the six rat groups, no transfer of adipocyte Glut4 and IR (Figure 11a,b) as well as erythrocyte Band-3 and Glycophorin (Figure 11c–f) was detected. Moreover, for each combination and serum incubation, final injection of PI-PLC (at 6200–6500 s) resulted in decrease of GPI-AP transfer (at 6200 s) by 50 to 85%. This reemphasized the efficacy of the transfer for GPI-APs compared to transmembrane proteins.

Quantitative evaluation revealed drastic differences in the transfer of total GPI-APs in the presence of serum proteins between the various donor–acceptor PM combinations with identical ranking for each rat group with decreasing efficacy in that order (Figure 11): hE → rE > rE → hA > rE → hE > rE → rA > hA → rE > rA → rE. These data confirmed the above finding (see Figure 7) that the transfer efficacy is determined by both donor and acceptor PM.

Most importantly, significant differences in GPI-AP transfer became apparent between the six rat sera, which were independent of the donor–acceptor PM combination (Figure 12a). Consequently, maximal differentiation power was obtained by summing-up the phase shift differences measured for all six donor–acceptor PM combinations for each of the six rat groups and calculating the % inhibition of GPI-AP transfer (Figure 12b). This resulted in significant differences between the six rat groups with increasing transfer inhibition in that ranking order: lean Wistar < ZF < ZDF < obese Wistar < ZF < ZDF.

The differential inhibition of GPI-AP transfer by serum proteins from rats of different metabolic phenotype may be explained by subtle differences in the steady-state and kinetic parameters of their binding to the GPI anchor of GPI-APs, such as affinity and k_on_- and k_off_-rates. Those could be rate-limiting for the relief of serum proteins from binding to GPI-APs, and thus for their subsequent translocation into the PM of tissue and blood cells in vivo.

## 4. Discussion

### 4.1. Cell-Free Analysis of the Intercellular Transfer of GPI-APs

The major advantage of studying cellular processes with cell-free assays, in general, relies on the use of defined molecular components and experimental conditions as well as on their straightforward manipulation with the aim to identify the optimal configuration, which may also be relevant in vivo. In particular, cell-free assaying of the intercellular transfer of GPI-APs with the aid of a microfluidic chip-based SAW sensor, as introduced in the present study, enables the variation of the donor and acceptor PM derived from relevant tissue and blood cells, such as adipocytes and erythrocytes, at six different combinations as well as of the extracellular milieu, such as serum proteins, among them GPLD1. For this, acceptor PM covalently captured by the TiO_2_ chip surface (Figure 1a and Figure 2) were incubated with injected donor PM within the chip channels. After removal of the donor PM, the acceptor PM were assayed for the presence of GPI-APs and transmembrane proteins putatively transferred from the donor PM by injection of relevant antibodies (Figure 1b). Mass loading onto the chip surface accomplished (to a lower extent) by the transferred proteins per se and (to a higher extent) by bound antibodies (Figure 3) rather than (Ca^2+^-mediated) fusion of donor and acceptor PM (which was distinguished from transfer by kinetic and biochemical criteria; Figure 4 and Figure 5) led to right-ward shifts of the phase (phase shift increases) of the SAW which (as summation signal) reflected the transfer of proteins from donor to acceptor PM.

The data generated with the chip-based SAW sensing demonstrated that (i) rat and human adipocyte and erythrocyte PM can serve as both donor and acceptor for the transfer of GPI-APs (Figure 3 and Figure 6), (ii) transmembrane proteins do not undergo transfer to any detectable extent (Figure 3 and Figure 6), thus confirming previous findings [19,20,21,22], (iii) transfer efficacies differ between rat and human adipocyte and erythrocyte PM being highest between erythrocytes (Table 1), (iv) both donor and acceptor PM determine transfer efficacy (Figure 3 and Figure 6), compatible with release of GPI-APs from donor PM as well as their translocation into acceptor PM being of comparable importance for transfer, (v) transfer of GPI-APs is affected by the incubation conditions (Figure 4) and the milieu surrounding the donor and acceptor PM with serum proteins, downregulating its efficacy (Figure 8), (vi) interaction of the core glycan of the anchor of GPI-APs with serum proteins, such as GPLD1 (in particular in the inhibited state) or α-toxin, causes lowering of transfer efficacy (Figure 8 and Figure 9), suggesting that this action mode mediates (part of) the inhibitory effect of serum proteins and (vii) transfer involves the incorporation of full-length, but not of anchor-less GPI-APs or transmembrane proteins, together with annexin-V and cholesterol into micelle-like complexes (Figure 9 and Figure 10) rather than into membrane-/vesicle-like or lipoprotein-like structures (Figure 2e,f).

### 4.2. The (Patho)Physiological Relevance of the Intercellular Transfer of GPI-APs

In addition to the elucidation of the molecular components involved in and the biochemical conditions supporting the transfer of GPI-APs between cells of neighboring or distant tissue depots or compartments, the cell-free assay was helpful to obtain initial hints for the elucidation of the cellular function and (patho)physiological role of GPI-AP transfer in vivo, according to the following considerations:

The demonstrated transfer of full-length GPI-APs between adipocyte and erythrocyte PM, as well as between erythrocyte PM in both directions in vitro (Table 1; the transfer between adipocytes, could not be assayed due to non-availability of species-specific antibodies and similar levels of AChE as well as TNAP expression in rat and human adipocytes). This suggests operation in vivo of GPI-AP transfer between cells of different types, such as adipocytes, endothelial cells, and macrophages of the same adipose tissue depot via a paracrine route, or adipose tissue cells and blood cells via an endocrine route as well as between cells of the same type, such as erythrocytes, via an endocrine route. Given the well-documented advantages and disadvantages of GPI anchorage of ectoproteins, such as maintenance of the biological function of the protein moiety [20,64,65,66,67,68,69] and membrane disturbance and lytic effects of the GPI moiety [32], respectively, it is tempting to speculate about GPI-AP transfer as a two-sided sword in the control of cell surface expression: Wanted within a tissue depot for the sake of compensation for insufficient expression at neighboring cells and unwanted between different tissue depots or blood compartment.

The decision between the putatively wanted functional or physiological paracrine transfer route and the unwanted non-functional/physiological endocrine route, made by a given GPI-AP, may be determined by the local arrangement of putative donor and acceptor cells within a tissue depot. In addition, limited accessibility of the interstitial spaces for inhibitory serum proteins and long distance between different tissue depots, as well as the presence of serum proteins, such as GPLD1, in the blood compartment may contribute to facilitation and impairment of transfer, respectively, i.e., to paracrine vs. endocrine routing of GPI-APs.

Proteins and factors have been identified which trigger down- or upregulation of transfer and apparently either interact with the core glycan of the GPI anchor, such as GPLD1 and bacterial α-toxin or interfere with this interaction, such as synthetic PIG, respectively (Figure 8 and Figure 9). This argues that intercellular transfer of GPI-APs is a regulated rather than spontaneous process as has already been suggested previously [70].

### 4.3. Metabolic Diseases and the Intercellular Transfer of GPI-APs

Strikingly, efficacy of transfer in the absence of serum proteins (Figure 6 and Figure 7) and inhibition of transfer by serum proteins (Figure 11 and Figure 12) were found to depend on the metabolic state of the rats providing the donor/acceptor PM and serum samples, respectively. Both turned out to be highest for hyperglycemia/hyperinsulinemia (obese diabetic ZDF rats), lowest for normoglycemia/normoinsulinemia (lean Wistar rats), and intermediary for normoglycemia/hyperinsulinemia depending on the plasma insulin level (Table 2) with the following ranking order of declining efficacy/inhibition: Obese ZF rats > obese Wistar > lean ZDF > lean ZF (Figure 7b and Figure 12b).

The apparent link between transfer efficacy and transfer inhibition may be explained as follows: 1. Specific alterations of the biophysical and biochemical properties of the PM in response to elevated blood glucose and plasma insulin favor release of GPI-APs from PM of tissue and blood cells, such as adipocytes and erythrocytes, and/or their translocation into PM and thus stimulate “overall” transfer. 2. Stimulation of transfer is paralleled by upregulation of expression of serum proteins, such as GPLD1, which prevent translocation of GPI-APs into PM, presumably by interaction with the core glycan of the GPI anchor. 3. The known deleterious effects of full-length GPI-APs and GPI anchors on the integrity of phospholipid bilayers of cultured cells [32] necessitate tight control of the transfer efficacy of GPI-APs, e.g., during hyperglycemic/hyperinsulinemic state, to ensure physiological function and viability of the acceptor cells. These explanations reinforce the value of a cell-free assay based on defined components (donor and acceptor PM, absence or presence of serum proteins) since in vivo the apparent counterregulation of stimulation and inhibition of transfer of GPI-APs by the obese/diabetic state would have resulted in steady-state level of transfer and thereby masked the role of the metabolic genotype and feeding state in transfer. The possibility of operation in vivo of intercellular transfer of GPI-APs, e.g., from adipocytes to erythrocytes, and of its mechanistic coupling to the metabolic state justifies future investigations for delineation of cause or consequence as well as of the potential for novel approaches for the prediction or cure of metabolic diseases, such as obesity and diabetes.

With regard to the apparent correlation of the efficacy of transfer of specific GPI-APs, i.e., of TNAP, CD73, AChE, CD55, and CD59, between adipocyte and erythrocyte PM and the metabolic state of the rats (diabetic/obese vs. healthy) as revealed in the present study, only CD73 has been linked to the regulation of glucose and lipid metabolism so far. The 5′-nucleotidase activity of CD73 converts extracellular AMP to adenosine [71,72], which is known to block lipolysis and contribute to diabetic insulin resistance via signaling through adenosine A_2B_ receptors [73]. In agreement, CD73-derived extracellular adenosine manages to control body fat homeostasis since deletion of CD73 has been reported to foster dyslipidemia and intramyocellular lipid accumulation in muscle of mice [74]. In particular, CD73 KO mice gained significantly less body weight and displayed lowered number and size of white adipocytes as well as increased serum free fatty acid and triglyceride levels compared to wildtype mice. This phenotype was accompanied by elevated blood glucose and serum insulin levels and impaired insulin signaling in skeletal muscle of CD73 KO mice, as reflected in decreased insulin-induced Akt phosphorylation. Insulin secretion and the level of insulin-degrading enzyme remained unaltered [74].

Interestingly, CD73 harboring the complete GPI anchor was reported to be released from cultured and primary adipocytes in microvesicles in response to metabolically relevant stress factors, such as high levels of palmitate, reactive oxygen species, and anti-diabetic drugs [75,76,77,78]. Furthermore, the level of CD73 in plasma was shown to be correlated with insulin sensitivity in diabetic mice and human probands [79,80,81,82].

In addition to CD73, only a few other GPI-APs have been linked so far to glucose and lipid metabolism, among them glycolipid-anchored cAMP-binding ectoprotein (Gce1), T-cadherin, and glypican-4 (Gpc4). Gce1, which binds and cleaves cyclic adenosine monophosphate (cAMP) through phosphodiesterase activity, has first been identified at the outer leaflet of PM of yeast [83] and then rat adipocytes [38]. Gce1 cooperates with CD73 in the degradation of cAMP via AMP to adenosine [84]. Both are thought to coordinate the inverse regulation of lipid degradation and synthesis at the surface of intracellular lipid droplets between small and large adipocytes [85,86].

T-cadherin acts as a GPI-anchored cell surface coreceptor [87] for the hexameric and high-molecular-weight species of adiponectin [88]. This adipokine is exclusively secreted by differentiated adipocytes [89] and is downregulated in the serum of obese and diabetic rodents and humans [90]. Since those adiponectin species have been demonstrated to activate NF-κB [91], T-cadherin expressed in endothelial and smooth muscle cells has been linked to the anti-inflammatory response of adiponectin in course of metabolic syndrome and endothelial dysfunction [92]. It remains to be investigated whether GPI-anchored T-cadherin is transferred from those cells to adiponectin effector cells which display low T-cadherin expression, such as myocytes and hepatocytes. In this case, transfer may contribute to adiponectin-induced stimulation of fatty acid oxidation in muscle and glycogen synthesis in liver as well as inhibition of gluconeogenesis in liver [93].

Gpc4 is a member of the family of GPI-anchored heparan sulfate proteoglycans and supports as a coreceptor a number of growth factors, such as Wnt, fibroblast growth factors, and Hedgehog in mammals [94,95]. Gpc4 was reported to regulate insulin signaling via interaction with the insulin receptor [96]. Importantly, both membrane-associated GPI-anchored and soluble anchor-less Gpc4 were able to interact with the unoccupied insulin receptor and to stimulate insulin signaling, whereas the occupied insulin receptor failed to interact with Gpc4. Overexpression of the native GPI-anchored Gpc4 in or incubation of the recombinant anchor-less Gpc4 with 3T3-L1 adipocytes caused upregulation of insulin signaling, whereas depletion of Gpc4 blocked insulin receptor autophosphorylation and downstream signaling [96].

Interestingly, Gpc4 was detected in serum of mice and humans, with levels being positively correlated to body fat mass and insulin resistance [96]. The expression of soluble Gpc4 in serum and its relationship to BMI and glucose tolerance could rely on its lipolytic release from the surface of donor cells. In fact, GPI-specific phospholipases C and D were demonstrated to cleave the GPI anchor of Gpc4 [97,98]. Furthermore, serum levels of GPLD1 were shown to be elevated in response to feeding a high-sucrose diet [99], but to be diminished in *ob*/*ob* mice [100] as holds true for Gpc4 [96]. The strong correlation between serum Gpc4 levels and BMI in humans together with the observation that Gpc4 is released from primary adipocytes in vitro strongly argue for adipose tissue as the major source of serum Gpc4. These findings have been interpreted to indicate that Gpc4 acts as an insulin-sensitizing adipokine by direct interaction with the insulin receptor and accompanying activation and downstream signaling independent of whether being presented in the GPI-anchored or soluble lipolytically cleaved version.

The data presented in this study now raise the possibility that (part of) the link between glucose/lipid metabolism and the function of certain GPI-APs previously attributed to their stable surface expression at certain cell types, such as adipocytes [74,96,101,102,103,104,105], or to their cleavage into a soluble anchor-less version [97,98,99,100] relies on the paracrine or endocrine transfer of their full-length versions from donor to acceptor/effector cells.

### 4.4. Future Studies of Intercellular Transfer of GPI-APs In Vivo

The presented findings about stimulatory and inhibitory factors of transfer of GPI-APs between PM in vitro should motivate analysis of the (patho)physiological relevance of intercellular transfer in appropriate animal models for obesity and diabetes. One option relies on the expression of green fluorescent protein (GFP) as GPI-anchored version (GPI-GFP) in relevant tissues, such as adipose, liver, and muscle, in transgenic healthy, obese, and diabetic mice using tissue-specific inducible promoters. The route of GPI-GFP from expressing to non-expressing cells of the same tissue depot (paracrine route) or of different tissue depots (endocrine route) may be determined by high-resolution imaging at various time points upon induction. Moreover, this technology would enable the investigation of intercellular transfer of GPI-GFP in response to endogenous (genotypic) and/or exogenous (environmental) cues, such as ageing, nutritional state, and stress. Thereby, the possibility of control of expression of cell surface proteins is not solely determined by gene expression in the corresponding cell type but, in addition, by acquisition of GPI-APs from neighboring or distant tissue and blood cells upon transfer through direct contact or via body fluids would be addressed.

Considering physiological relevance, it may be of interest to see whether transfer of GPI-APs is confined to certain microdomains (lipid rafts) of the acceptor PM [106,107]. In non-polarized cells, such as fibroblasts and T-cells, GPI-APs are organized in cholesterol-containing nanoclusters [108]. At variance in polarized epithelial cells, such as Madin-Darby canine kidney and intestinal cells, GPI-APs of a single species initially become targeted to small cholesterol-independent homoclusters, which subsequently coalesce into larger cholesterol-dependent heteroclusters consisting of multiple GPI-APs species [109,110]. Moreover, it has been demonstrated previously that in fully polarized cells, GPI-APs are directly sorted to the apical cell surface without passing through the basolateral PM. This argues for apical vs. basolateral sorting of GPI-APs at intracellular sites prior to arrival at PM [111,112]. Thus, considering transfer of GPI-GFP to PM during cellular or animal studies, several possibilities are conceivable for the final targeting/destination of transferred GPI-GFP: Homogenous distribution over the complete PM vs. clustering in microdomains and, in addition, in polarized cells, exclusive expression at either the apical or the basolateral surface vs. uniform distribution over the complete cell surface [113]. In any case, the recently demonstrated impact of different carboxy-terminal GPI-attachment signals on apical vs. basolateral trafficking of GPI-APs via control of their oligomerization state [114] has to be considered for the construction of GPI-GFP passenger candidates suitable for studying intercellular GPI-AP transfer in vivo.

After successful visualization of donor and acceptor cells fostering GPI-AP transfer via the paracrine or endocrine route, the nature of GPI-APs specifically transferred in course of a given (patho)physiological state should be identified. With this information, the causal relationship between the paracrine or endocrine transfer of specific GPI-APs and a normal or disease phenotype may be studied in mice with knockout/in of the genes encoding the authentic GPI-AP/chimeric transmembrane version, which have to be constructed by exchange of the signals for GPI and transmembrane anchorage [115,116,117].

### 4.5. Conclusions

The cell-free chip-based sensing assay for the transfer of full-length GPI-anchored cell surface proteins between PM, introduced in the present study (for human and rat erythrocytes and adipocytes), demonstrated its dependence on the metabolic state (here obese and diabetic) of the donor organism (here rats) and its control by serum proteins (here in particular GPLD1). Upregulation of transfer by hyperglycemia and hyperinsulinemia is counterbalanced by serum proteins, which interact with the GPI anchor of the cell surface proteins within micelle-like complexes upon release from PM. This assay will be helpful for identification of the components, tissues, and (patho)physiological processes specifically involved in intercellular transfer of cell surface proteins as well as for screening for drug candidates which modulate transfer in course of dysregulation as cause for or consequence of certain (metabolic) diseases.

The available experimental body of evidence clearly indicates that intercellular transfer of GPI-APs via non-membrane structures, i.e., micelle-like GPI-AP complexes [30,31,32,33] or lipoprotein-like particles [29,58,118,119,120], as analyzed in the present study, must be regarded as a mode of protein transfer between cells. Protein transfer has meanwhile gained acceptance as a mechanism for the regulation of the (surface) expression of a given protein in a given cell independent of the expression of the corresponding gene in that cell. Another mode is represented by extracellular vesicles which manage to transfer both membrane and soluble proteins in course of budding from donor cells and subsequent fusion with acceptor cells [1,2,3,4,5,6]. Recent studies have unequivocally demonstrated the (patho)physiological relevance of protein transfer in vivo, e.g., for the adipocyte-extracellular-vesicle-endothelium axis, which is governed by the metabolic state [121,122,123,124].

Finally, it has to be stated that intercellular transfer of GPI-APs does not represent the only path for a cell to get rid of GPI-APs, but rather is only a minor one. The others, identified during the last three decades, encompass (i) endocytosis followed by sorting to the endosomal/lysosomal compartment for recycling/degradation of unwanted/non-functional GPI-APs [125,126], (ii) in polarized cells, transcytosis following internalization from one PM compartment, targeting of transport vesicles across the cytoplasm and their fusion with the other compartment for trafficking of GPI-APs from the apical to the basolateral cell surface vice versa [127,128], (iii) shedding from the cell surface in course of lipolytic cleavage (see Introduction) for hepatic clearance or operation as signaling molecule or mediation of effects at distinct sites, (iv) release in extracellular vesicles (see Introduction) and (v) in lymphocytes trogocytosis as the extraction of GPI-APs embedded in intact PM fragments from the cell surface of antigen-presenting cells and subsequent transfer to T, B and natural killer cells [129,130,131]. This panel of possible fates and functions of GPI-APs upon expression at the cell surface has now to be supplemented with intercellular transfer via non-membrane structures corroborating the diversity and complexity of the biology and (patho)physiology of GPI-APs.

## Figures and Tables

**Table 2:** Characteristics of the six rat groups. Mean values ± SD (*n* = 8) of weight, fasting blood glucose and fasting plasma insulin for each rat group (of given genotype and feeding state) are shown (* *p* ≤ 0.01, ^§^ *p* ≤ 0.02, ^#^ *p* ≤ 0.05 vs. lean Wistar).

Genotype	Feeding State	Weight [g]	Age [week]	Fasting Blood Glucose [mM]	Fasting Plasma Insulin [µg/L]	Metabolic Phenotype
Wistar	lean	328.3 ± 40.2	10	6.58 ± 0.23	0.95 ± 0.19	normoglycemic normoinsulinemic
	obese	519.6 ± 59.2 *	10	7.07 ± 0.69	2.17 ± 0.38 *	normoglycemic mildly hyperinsulinemic
ZF	lean	481.5 ± 51.3 *	40	5.65 ± 0.44 ^#^	0.90 ± 0.26	normoglycemic normoinsulinemic
	obese	682.0 ± 74.9 *	40	5.61 ± 0.40 ^#^	3.39 ± 0.61 *	normoglycemic hyperinsulinemic
ZDF	lean	377.2 ± 43.8	16	6.05 ± 0.47	1.28 ± 0.29	normoglycemic mildly hyperinsulinemic
	obese	428.9 ± 55.9 ^§^	16	20.40 ± 1.23 *	2.35 ± 0.55 *	hyperglycemic hyperinsulinemic

## Data Availability

The datasets generated and analyzed during the current study are available from the corresponding author (G.A.M.; guenter.mueller@helmholtz-muenchen.de) on reasonable request and will be provided as the original SAW data files together with the appropriate SAW software for data visualization and processing, if required, under consideration of the relevant conditions for licensing of FitMaster®, SensMaster® and SequenceMaster®.

## Supplementary Materials

The following are available online at https://www.mdpi.com/article/10.3390/biomedicines9101452/s1, File S1: Fundamentals of biomolecule sensing with surface acoustic waves using the samX-biosensor from SAW Inc. (Bonn, Munich, Germany).
