# Supplementary material for: Chip-Based Sensing of the Intercellular Transfer of Cell Surface Proteins: Regulation by the Metabolic State
Source: Biomedicines. 2021 Oct 13;9(10):1452. doi: 10.3390/biomedicines9101452 (PMC8533487; doi:10.3390/biomedicines9101452)
Supplement: Supplementary file 1 [file biomedicines-09-01452-s001.zip › biomedicines-1386580-supplementary.pdf]

**S U P P L E M E N T A R Y   M A T E R I A L S**

## Fundamentals of Biomolecule Sensing with Surface Acoustic Waves Using the samX-Biosensor from SAW Inc. (Bonn, Munich, Germany)

### PRINCIPLE

**Measurement**

The measurement principle of the samX-biosensor is based on locally defined propagation of acoustic waves. Acoustic wave sensors are used for the detection of mass loading on the sensor surface as well as for the detection of fluidic properties like the density and viscosity of the product.

In general, quartz crystal microbalances (QCM) are of bulk acoustic wave ensor type or of surface acoustic wave sensor type (SAW). The SAW sensor type has the advantage of being smaller and more sensitive than bulk sensors. To acieve high sensitivity for biochemical binding experiments, the actual samX SAW sensor applies shear waves in an additional amorphous guiding layer (so-called Love waves). Thereby, the acoustic waves are only marginally damped in an aqueous environment. The guiding layer also provides high wave amplitudes at the sensor surface to enhance the overall sensitivity.

The actual samX SAW sensor chip arrays consist of four identical sensor elements with a sensitive area of 5 x 1.2 $mm^2$ per sensing element. They are fabricated with thin film and photolithography techniques. The main features of the array are:

a) Contact pads to the read-out electronics

b) Interdigital transducers (IDTs) transforming electrical signals into acoustic waves and vice versa

c) $SiO_2$ guiding layer. The material of the sensitive area between the IDTs can be customized. The standard material of the sensitive area of samX SAW sensor chips is a gold layer of 100 nm thickness.

The samX can hold one or two chips. This gives a high flexibility in system operation. Three different modes canbe applied:

1) One experiment with one chip, therefore four channels

2) One experiment with two chips, therefore eight channels

3) Two experiments with each one chip, therefore two times four channels.

A complex but fully automated fluidic unit enables the activation of one, two or four channels. If one experiment with two chips os selected, in addition all eight channels can be activated. This gives the possibility to e.g. immobilize up to eight different ligands, followed by a joint flow of a single analyte over the different surfaces. The illustration below exemplifies the experiments with one chip in the upper row and with two chips in the lower row.

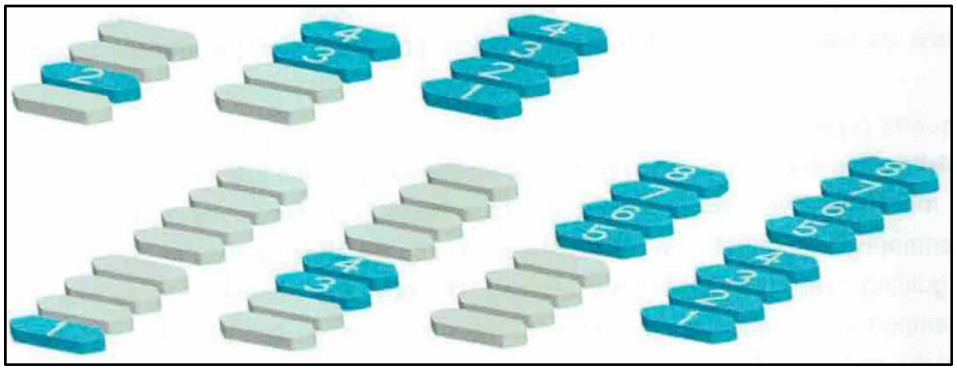

Furthermore, the regular buffer flow can be selected from two reservoirs. This enables to e.g. chemically immobilize the ligand in water, followed by biological interaction in a corresponding running buffer.

For biological experiments, receptor molecules (ligands) like antibodies, aptamers, DNA strands or whole cells can be used. Receptors can be immobilized onto the sensitive area by direct adsorption to the surface material, or via a spacer layer, which cann for example be a self-assembled monolayer (SAM) of an alkanethiol or carboxymethyl-dextran.

For correct interpretation of biological experiments, both the amplitude and the phase change between output and input signal are recored at two different frequencies. This is controlled by an integrated microprocessor connected to a PC, which operates as the user interface.

A multiplex circuit addresses the different sensor elements, while the read-out electronics is based on an I/Q-demodulator and a frequency synthesizer. This set-up allows the separation of mass and viscosity changes and the use of individual sensor elements as reference channels. Theoretical calculations show that an additional rigid mass on the sensor surface results in a pure phase shift proportional to the mass loading, while changes in the viscosity lead to a combined phase shift and change in amplitude. These experimentally proven dependencies are used for data evaluation in the samX software.

A two-frequency measurement mode is used to significantly reduce the inherently present sensitivity differences between the individual sensor elements. Cross sensitivities based on varying ion concentrations of the buffer are rejected. Highly comparative measurements are enabled. From a technical point of view, the measured signal can directly be evaluated without a reference sensor. A reference channel might be subtracted to correct for undesired binding based on the surface preparation.

**Chip-Based Sensing of Full-Length GPI-APs in Micelle-Like Complexes**

Specific capture of the GPI-APs by the chip-based SAW sensor is accomplished by binding to α-toxin (see figure below). Covalent coupling of α-toxin to the chip gold surface is performed using the conventional EDC/NHS-based protocol and monitored by measuring the phase shift in course of the reaction. Signals generated by the sensor and recorded in real-time reflect the loading of mass onto the chip surface and, in addition, depend on the (bio-)physical properties of the contacting sample fluid, predominanly its viscoelasticity. Capture of the unprocessed GPI-APs leads to right-ward shifts in phase and/or reductions in amplitude of the shear-horizontal SAW propagating along the chip surface (green curve) *vs*. blank chip (black curve). Phospholipids in complex with the unprocessed GPI-APs become detected in course of sequential binding "in sandwich" of the $Ca^{2+}$-dependent phospholipid-sequestering protein annexin-V which leads to further right-ward shift in phase and reduction in amplitude (blue curve) *vs*. blank chip (black curve) as indicated by brown and orange triangles/arrows, respectively.

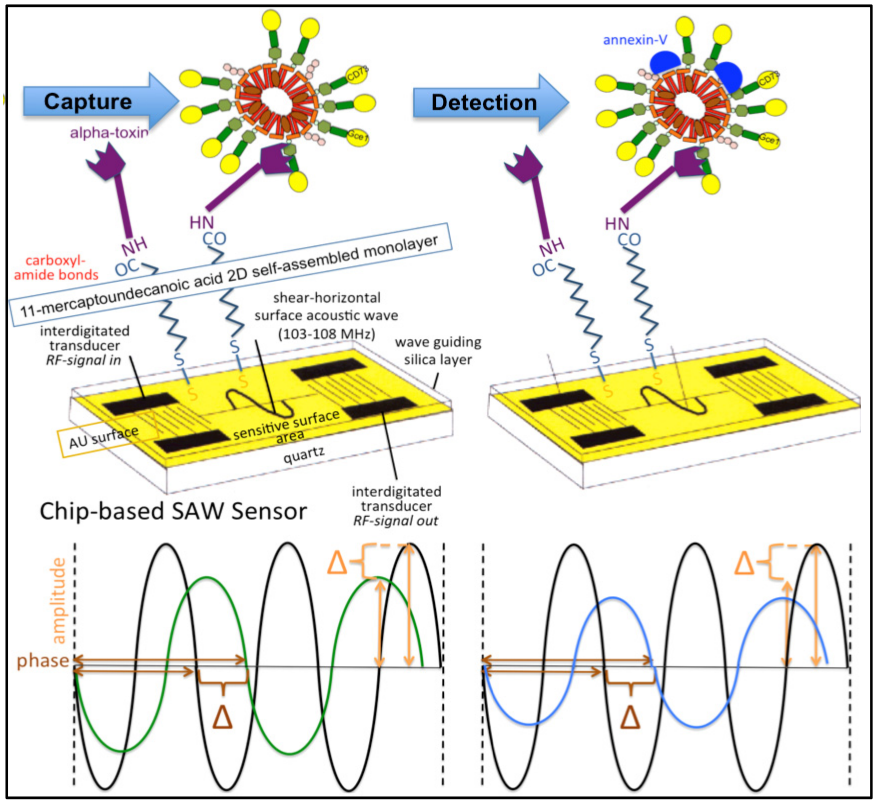


## THE SYSTEM

The samX system consists of hardware and software components. To take full advantage of the system, it is necessary to understand the tasks of the single components and their interactions.

The central components of the system is the samX sensor unit. It provides the connection of the sensor chip to the fluidic system and processes the electrical signals from the sensor chip. The standard samX sensor unit incorporates one pump providing a continuous flow of buffer or analayte over the sensor chip. The pump is connected via internal valves to the two fluidic cells in the top cover of the samX sensor unit. An autosampler is integrated into this flow path, enabling injection of analytes into the constant buffer flow. (In the following, the term 'measurement' refers to the period of time during which the sensor signal is detected. The term 'sequence' refers to a series of commands, which e.g. control the flow rate of the pump, the injection of samples, switching of active channels and waiting times between injections)

**samX System**

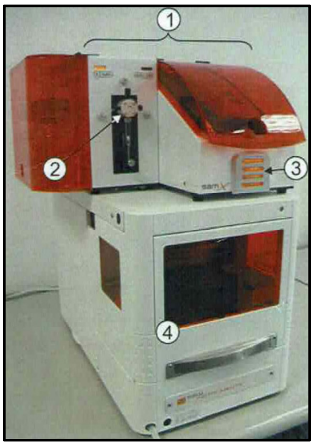

The samX sensor unit **(1)** is the core of the complete samX system. It contains most components of the system, i.e. the complete electronics, RF (radio frequency), power and control unit. Next to the chip reader provides a single syringe pump **(2)** a continuous liquid flow over the sensor. Connections for power supply and communication between PC and autosample are on the back side of the sensor unit. Four LED panels indicate the status of the system **(3)**. The autosampler **(4)** is located below the chip reader for automated injections.

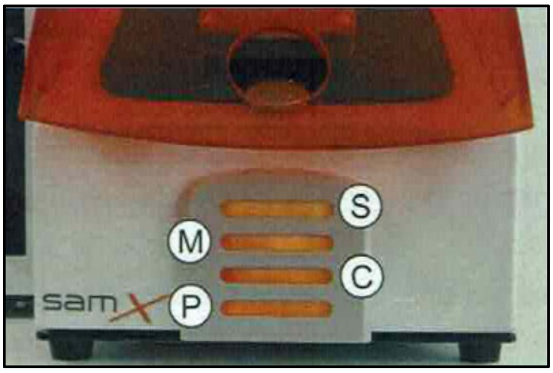


The LEDs indicate the following states of the system (from bottom to top):
- Power on (P)
- Chips calibrated (C)
- Measurement running (M)
- Sequence running (S)

**samX Sensor Unit**

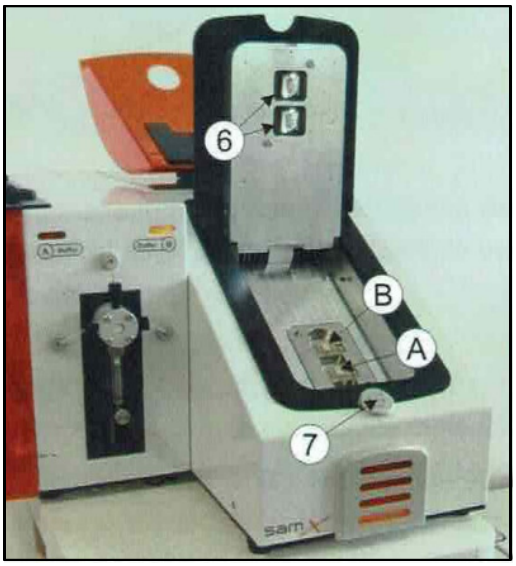


Within the samX sensor unit, the two sensor chips in the **(A)** front and **(B)** back are electrically and fluidically connected to the read-out electronics of the chip reader. The electrical contacts as well as the fluidic cell are integrated within the top part of the unit **(6)**. The orange cover is used for thermal isolation. The sensor unit can be unlocked by pushing the opening button **(7)** to insert the sensor chip carrier in the corresponding uptake.

**Sensor Chip and Chip Carrier**

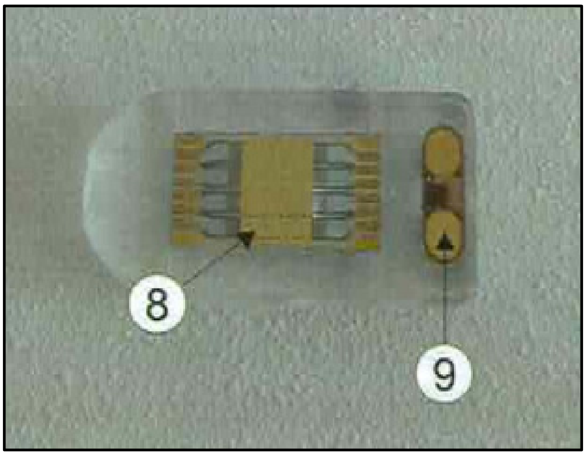


The sensor chip is fixed into a chip carrier. This ensures an exact positioning of the sensor chip within the sensor unit. The 15 x 8.5 $mm^2$ sensor chip contains four single sensor elements. The sensitive area of a single sensor element is covered by a top layer of gold **(8)**. In addition, a memory-device **(9)** is placed on the right side of the chip. It stores expiration-date, initial coating and labels / names of the different sensor elements.

## FLUIDICS

The fluidic system mainly consists of the following components: autosampler, syringe pump, bottle holder with fluidic interconnects, internal valves, and two fluidic cells.

**Autosampler**

By means of an autosampler, samples are accurately injected into the active channels of the fluidic cells. Injections are defined in the *SequenceMaster* program, permitting unattended operation. The autosampler is controlled by the chip reader unit via a serial link. The order of injections is defined by the user sequence.

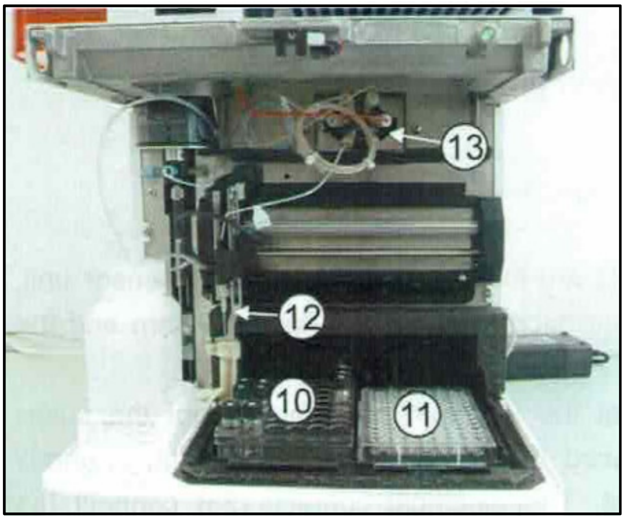

The autosampler has two tray positions (left and right), which holds a removable vial tray **(10)**, or a 96-well microtiter-plate **(11)**. After a sample is taken up by the needle **(12)**, it is injected into the continuous buffer flow by the injection valve **(13)**.

**Pump**

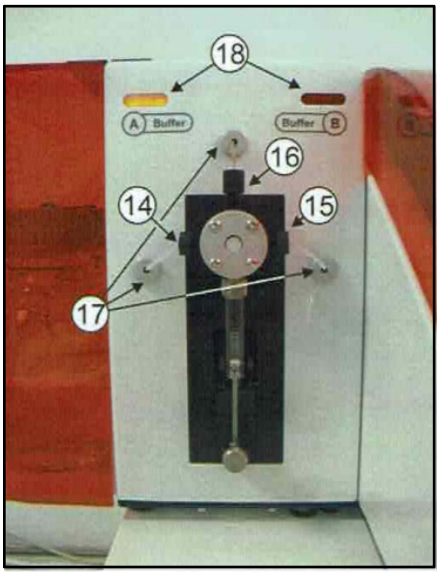


The syringe pump at the front side of the samX sensor unit is required for continuous flow of buffer solution and sample transport within the fluidic system. At the top, the pump provides three connectors for fluid tubing:
**(14)** The left one serves as inlet for buffer A.
**(15)** The right one serves as inlet for buffer B.
**(16)** The upper one connects to the autosampler.
Fluidic interconnects **(17)** guide the tubing through the system. LED panels **(18)** indicate, which buffer is currently used within the system.

**Bottle Holder with Fluidic Interconnects**

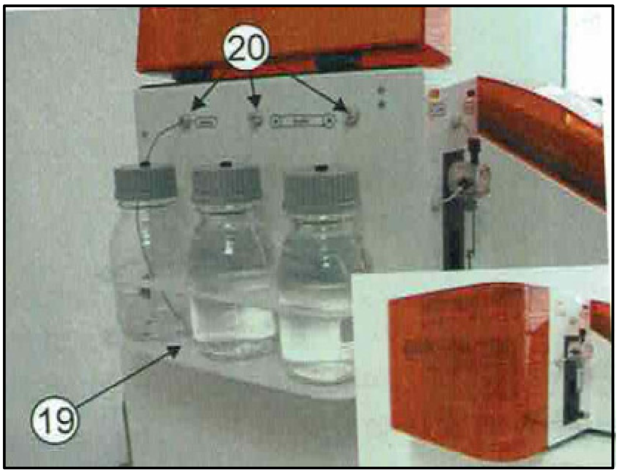

The bottle holder **(19)** is mounted to the left of the pump and holds three 250-mL bottles. The central bottle is designed for the buffer a reservoir, the front bottle is used for the buffer B reservoir, while the bottle for waste is located at the rear. Corresponding labels indicate their uasage. Fluidic interconnects **(20)** guide the tubing through the system. The inset shows the bottle holder with closed cover.

**Fluidic Cells**

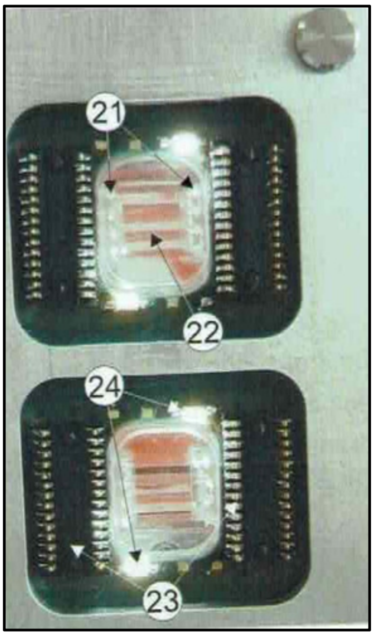


Two fluidic cells are located at the top of the sensor unit, providing the interface between the fluidic system and the sensor chip. Tubings **(21)** at the left and the right side of the fluidic cell **(22)** are used to individually address each channel/sensor element. The electrical contacts **(23)** connect the sensor chip to the electronics. LEDs **(24)** for illumination of the chip and the fluidic cell can be switched on or off on the backside of the samX.

## LIQUID HANDLING

**Regular Flow**

Constant flow is established by means of the syringe pump. The selected buffer (A or B as indicated by the LED) will be aspirated from the pump and injected into the sensor unit. With the option *Pump/Change Buffer*, the buffer in the system can be exchanged. As all tubings need to be rinsed, this change might take several minutes. The menu option *Pump/Start* runs the selected buffer continuously through the sensor unit. In the standard setup, a range from 12.5 to 300 µL/min can be applied. The buffer flows via the autosampler to the sensor unit, allowing injections of different samples. The current flow rate is indicated at the bottom of the SensMaster software. It might be changed by the menu option *Pump/Start*.

The following flow chart shows the way of the buffer:

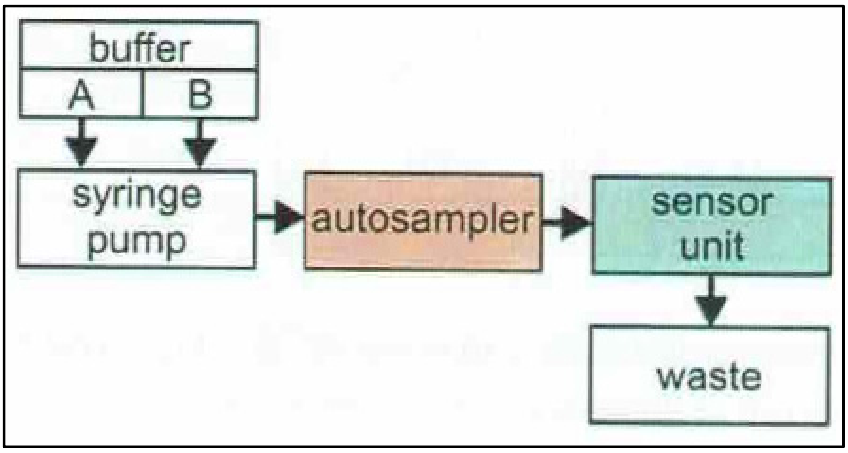


**Injection of Samples**

The autosampler (AS) is used to inject a sample into the regular flow. The general programming of the injections is described in the *Sequence/Master* manual.

By use of the following two schemes, the flow paths in standard applications are visualized. In this setup, the sample is placed into a vial or microtiter plate. During the loading process, the sample is aspirated with the needle from the vial into the sample loop using the internal AS pump (blue path).

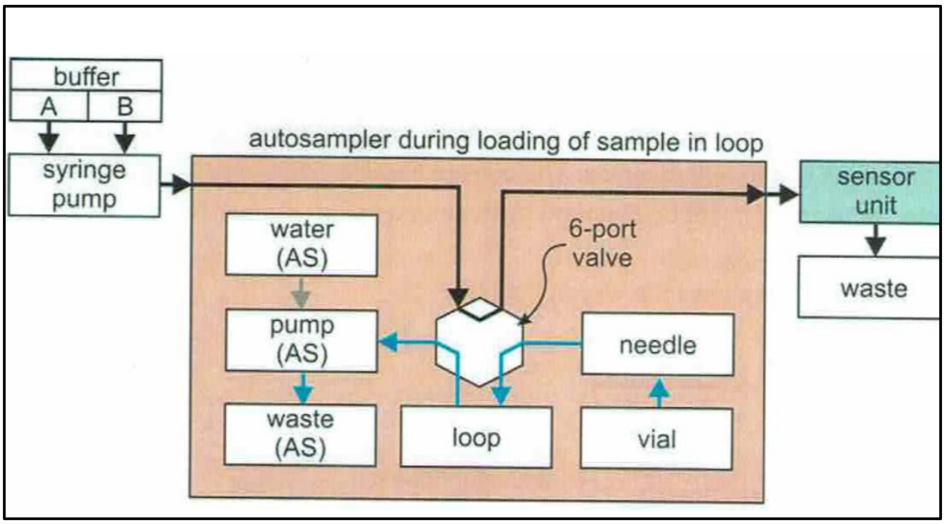


Afterwards, the 6-port valve is switched to the injection position. The sample is injected into the sensor unit by the external pump (red path). At the same time, the needle is cleaned with water from a reservoir within the AS, using the internal pump (green path).

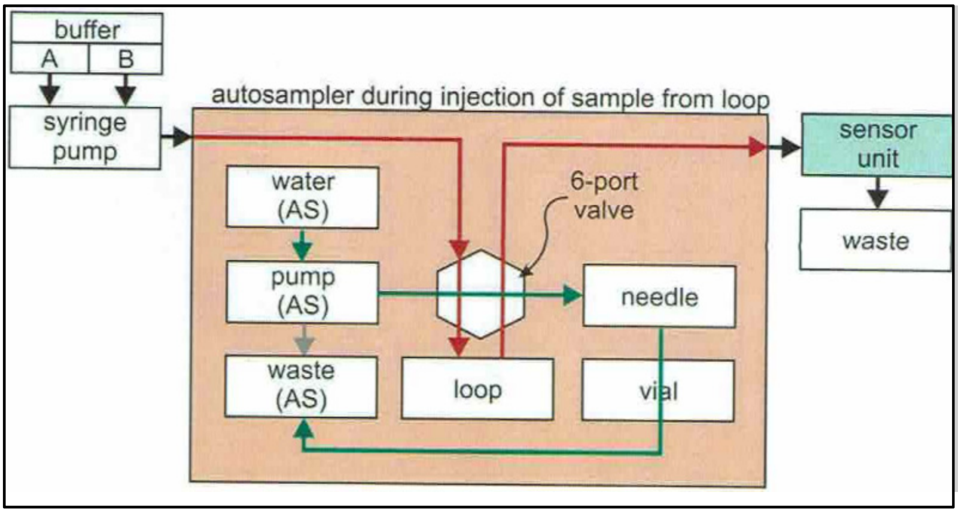


**Sample and Buffer Handling**

To avoid the appearance of disturbing gas bubbles during a measurement, the buffer solution has to be degassed carefully.

A supplemental specific waste volume is added to the sample volume to avoid bubbles within the fluidic system. The acual waste volume differs depending on the type of vial used. Vial type and total volume are automatically recommended for each injection in the *SequenceMaster* software. Only vials with a soft cap should be applied, since standard plastic vials might damage the needle. If possible, vials should be centrifuged to degas contained samples and to minimize losses.

**Selection of Active Channels**

Within the sensor unit, internal valves can adress the individual channels of the two fluidic cells. One, two, four, or eight channels can be used. The continuous flow runs only over the active channels, while the flow is stopped on the remaining channels. Accordingly, the sample from the autosampler is only injected over the sensitive area of the active channels.

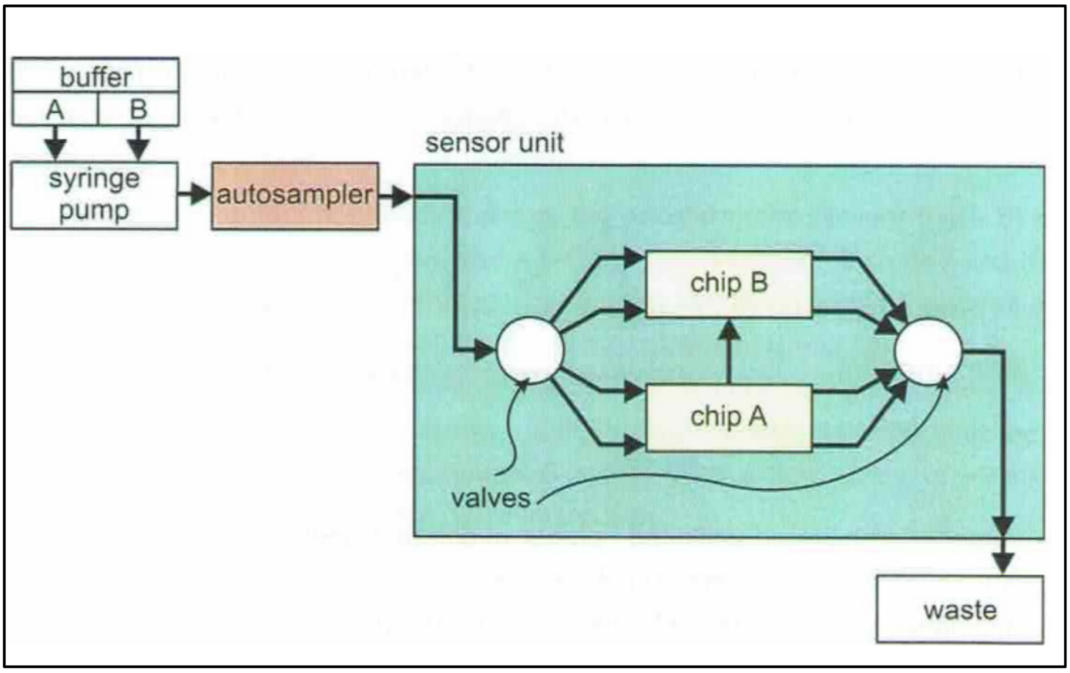

## MEASUREMENT-CONCEPT

In order to understand the measurement-principle of the samX, it is important to differentiate between a measurement, a sequence and injections.

### Measurement

A measurement is the continuous collection of data. It can be started by pushing the start button **(1)**. The collection of data stops by pushing the stopp button **(2)**.

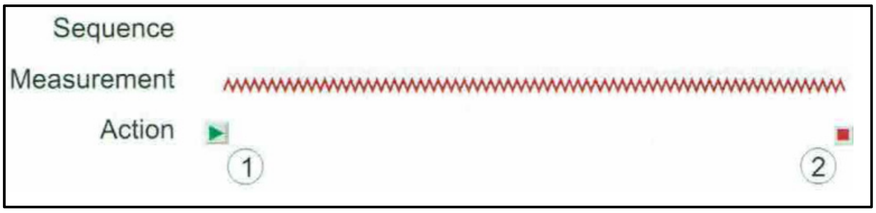


### Sequence

A sequence controls the fluidic unit including the unlimited number of injections of samples from the autosampler **(4)**, changes in flow-rate, changes in active channels, or the active buffer.

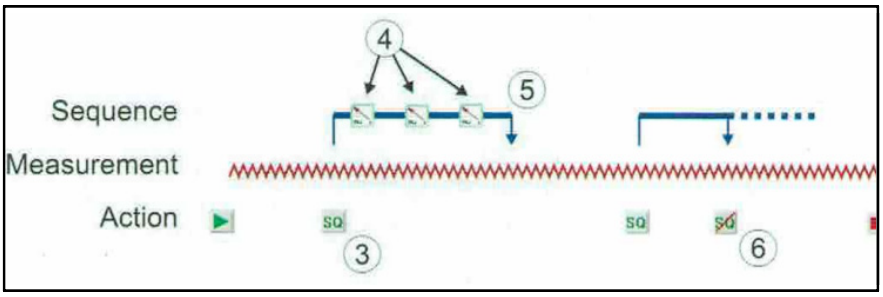


A measurement can have an unlimited number of different sequences. A sequence can be started with a start sequence button **(3)**. After execution of all injections within the sequence, it stops automatically without user interaction **(5)**. Then, another sequence can be started. If a sequence does not work properly, it can be aborted with a stop sequence button **(6)**. As long as the measurement is still running, further sequences can be initiated.

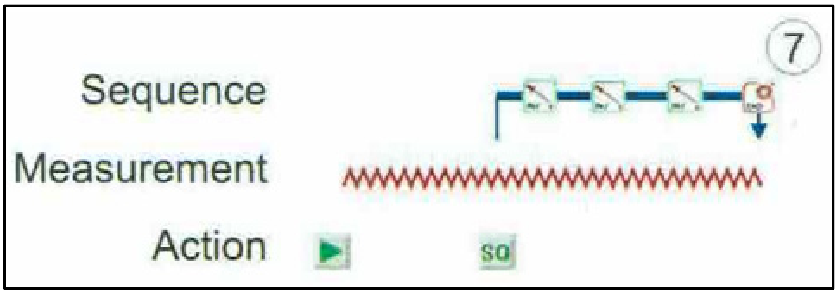


A measurement can be automatically terminated after completion of a sequence (e.g. during night or weekend operation). The integration of an end-command **(7)** as last command of a sequence stops the measurement. In this case, no further sequences can be added to the measurement. Alternatively, the buffer flow can be stopped altogether or reduced, at least.

**Experiment A➔**

The experimental concept A➔B enables the user to use the samX as if it had two independent sensor units, each with a single chip. Therefore, two measurement-windows with four channels open. Only one measurement can be performed at a time. The second measurement will then start after the first measurement has finished.

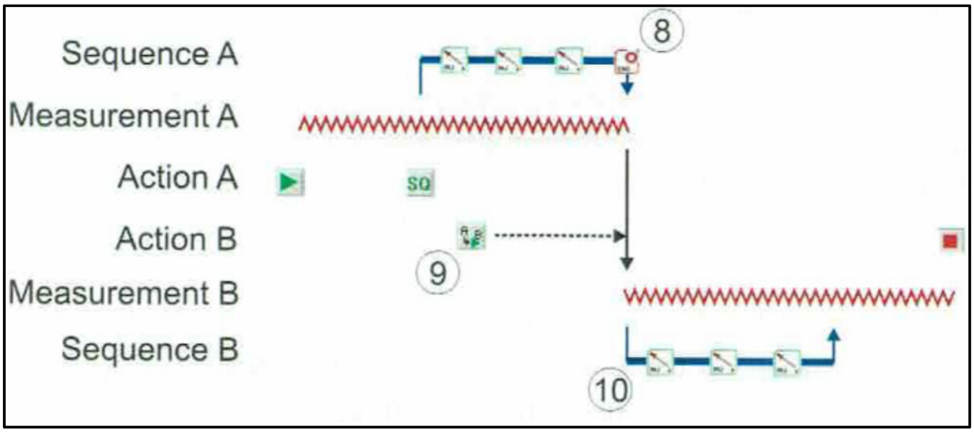


The first measurement (here A) can be started as a regular measurement: At first, measurement has to be started, then sequence. However, the second measurement only starts automatically when the first measurement is terminated after the last injection by an end command in sequence A **(8)**. While the first measurement is already running, the second measurement (here B) can be prepared. Unlike in the regular process where first the measurement is started and then the sequence, only one spepcific button is used. This button A➔B **(9)** within the measurement window of the second measurement will start both measurement B and sequence B.

However, as measurement A is still running, this process will not start immediately. It is kept on hold (indicated by the dashed arrow), until the measurement A has finished. After completion of measurement A, measurement B starts. With a time delay of about 2 min, also sequence B will start **(10)** without further user interaction. Measurement B will continue to collect data, until it has been stopped manually, or by an end-command integrated into sequence B.
